# Context-specific action of macrolide antibiotics on the eukaryotic ribosome

**DOI:** 10.1038/s41467-021-23068-1

**Published:** 2021-05-14

**Authors:** Maxim S. Svetlov, Timm O. Koller, Sezen Meydan, Vaishnavi Shankar, Dorota Klepacki, Norbert Polacek, Nicholas R. Guydosh, Nora Vázquez-Laslop, Daniel N. Wilson, Alexander S. Mankin

**Affiliations:** 1 Center for Biomolecular Sciences, University of Illinois at Chicago, Chicago, IL USA; 2 Department of Pharmaceutical Sciences, University of Illinois at Chicago, Chicago, IL USA; 3 Institute for Biochemistry and Molecular Biology, University of Hamburg, Hamburg, Germany; 4 Laboratory of Biochemistry and Genetics, National Institute of Diabetes and Digestive and Kidney Diseases, NIH, Bethesda, MD USA; 5 Postdoctoral Research Associate Training Program, National Institute of General Medical Sciences, National Institutes of Health, Bethesda, MD USA; 6 Department of Chemistry and Biochemistry, University of Bern, Bern, Switzerland

**Keywords:** RNA, Ribosome

## Abstract

Macrolide antibiotics bind in the nascent peptide exit tunnel of the bacterial ribosome and prevent polymerization of specific amino acid sequences, selectively inhibiting translation of a subset of proteins. Because preventing translation of individual proteins could be beneficial for the treatment of human diseases, we asked whether macrolides, if bound to the eukaryotic ribosome, would retain their context- and protein-specific action. By introducing a single mutation in rRNA, we rendered yeast *Saccharomyces cerevisiae* cells sensitive to macrolides. Cryo-EM structural analysis showed that the macrolide telithromycin binds in the tunnel of the engineered eukaryotic ribosome. Genome-wide analysis of cellular translation and biochemical studies demonstrated that the drug inhibits eukaryotic translation by preferentially stalling ribosomes at distinct sequence motifs. Context-specific action markedly depends on the macrolide structure. Eliminating macrolide-arrest motifs from a protein renders its translation macrolide-tolerant. Our data illuminate the prospects of adapting macrolides for protein-selective translation inhibition in eukaryotic cells.

## Abstract (web summary)

Macrolide antibiotics inhibit bacterial translation in a context-specific manner, arresting ribosomes at defined sites within mRNAs and selectively inhibiting synthesis of only a subset of cellular proteins. Here the authors provide a structural basis for the context-specific activity of macrolides on the eukaryotic ribosome.

## Introduction

Many human diseases result from expression of unwanted proteins^1–3^. While the most common therapies for such diseases are based on blocking the functions of the undesirable proteins, this approach mitigates their harmful effect but does not eliminate the culprit. Inhibiting the production of a malicious protein could be a better strategy than targeting its activity. To achieve this goal, significant efforts have been invested in developing mRNA-targeting approaches for selective destruction of specific mRNAs or blocking their translation^4^. However, very limited research has been dedicated towards finding molecules, that could curb the production of specific proteins by acting upon the ribosome. The lack of interest for undertaking such task could be justified by the traditional notion that ribosome-targeting compounds indiscriminately prevent ribosomes from synthesizing all proteins. Therefore, the discovery of PF846, a small molecule that binds to eukaryotic ribosomes and selectively inhibits translation of only a subset of polypeptides, including the therapeutically-significant protein PCSK9 involved in cholesterol homeostasis, came as a big and welcomed surprise^5,6^. Illuminating biochemical and structural studies have shown that PF846 binds in the nascent peptide exit tunnel (NPET) of the large ribosomal subunit and interferes with translation of several specific nascent polypeptides, that assume an idiosyncratic conformation in the tunnel^6,7^. However, because many details of the mechanism of PF846 action remain unknown, predicting the proteins whose synthesis would be inhibited by this compound would be a difficult task.

Even though the concept of selective inhibition of eukaryotic translation by ribosome-targeting small molecules emerged only recently, it has been long recognized for antibiotics that act upon the bacterial ribosome. Over four decades ago it was found that inducible resistance to macrolide antibiotics is regulated by programmed translation arrest relying on the ability of the drugs to stop ribosomes at specific mRNA codons, while allowing for unimpeded translation through the preceding ones^8,9^. More recent studies have shown that in fact many drugs that target the bacterial ribosome act in a context-specific manner, causing translation arrest at specific sites in mRNA, where the nascent peptide sequence, tRNA nature or mRNA structure are conducive to the antibiotic action (reviewed in the ref. ^10^). Yet, the ribosome-targeting antibiotics whose context-selective action is best understood are the macrolides^11^.

**Fig. 1:**
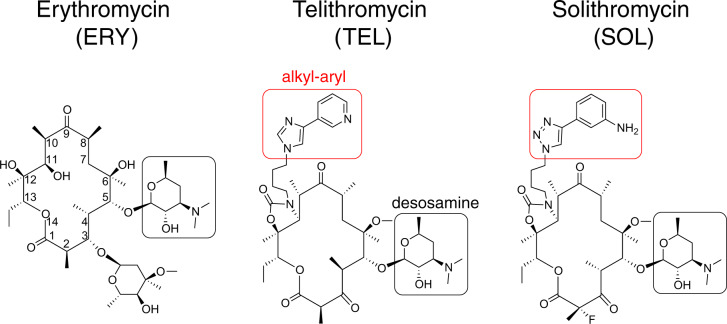
Macrolide antibiotics. Chemical structures of the main macrolide antibiotics used in this study. The numbering of the macrolactone atoms is shown on the ERY structure. C5-desosamine sugars and alkyl-aryl side chains of the extended macrolides are indicated by black and red rectangles, respectively.

All macrolide antibiotics, from the prototype of this class, erythromycin (ERY), to those of later generations, e.g., telithromycin (TEL) or solithromycin (SOL)^12^ (Fig. 1), bind in the NPET of the bacterial ribosome, close to the peptidyl transferase center (PTC)^13–16^. They establish interactions with several rRNA residues, including the adenosine at position 2058 of the 23S rRNA (*Escherichia coli* numbering) which, while conserved in bacteria, is replaced with guanine in eukaryotes (G2400 in the yeast *Saccharomyces cerevisiae* 25S rRNA) (Supplementary Fig. 1a, b), and is thought to be the key determinant of the bacterial selectivity of macrolide action^14^. Because the bulky macrolide molecule narrows the lumen of the NPET, these antibiotics were initially thought to completely block the passage of any nascent polypeptide and to only allow the synthesis of peptides a few amino acids long^17,18^. Subsequent studies have shown, however, that the growing protein chain can be threaded through the macrolide-obstructed NPET, and that macrolides selectively abolish production of specific proteins by preventing the ribosome from polymerizing specific amino acid sequences^11,19–22^. Remarkably, the context-specificity of the macrolide action is not based on the inability of some nascent polypeptides to bypass the antibiotic in the NPET. Instead, NPET-bound macrolides interfere with peptide bond formation when the bacterial ribosome attempts to synthesize specific amino acid motifs^23–27^. In addition, the spectrum of the macrolide arrest motifs, and hence the range of the inhibited proteins, is defined by the chemical structure of the drug bound in the NPET of the bacterial ribosome^11,19,22,28^.

Context-specificity and protein-selectivity of macrolide action in bacteria make this class of antibiotics a promising platform for developing selective inhibitors of eukaryotic translation. The prospect of such an approach hinges, however, on the macrolides retaining their context-selectivity and protein-selectivity of inhibition of protein synthesis in a eukaryotic cell. Unfortunately, little of what has been learned about macrolide action in bacteria could be extrapolated to eukaryotic translation, because the structures and functional properties of NPETs in bacterial and eukaryotic cytoplasmic ribosomes are substantially different^29–33^ (Supplementary Fig. 1b, c). Furthermore, whether macrolides can actually bind in the eukaryotic ribosomal NPET remains unclear, especially considering that mutation of the key discriminating nucleotide G2400 to A, failed to render yeast ribosomes or cells sensitive to ERY^34^.

Here, we used biochemical and structural analyses to explore whether macrolide antibiotics retain their inhibitory activity and context-specificity of action when bound to the eukaryotic (yeast) ribosome. We show that engineered yeast cytoplasmic ribosomes with the G2400A mutation are capable of binding macrolides, which contain an extended side chain and that the binding mode of such compounds in the NPET is analogous to that on the bacterial ribosome. We further show that macrolide binding to the 80S ribosome inhibits translation in vivo and in vitro. Genome-wide Ribo-seq studies revealed that in eukaryotic cells, macrolides interfere with protein synthesis in a context-specific way, with some of the prevalent arrest motifs overlapping with those in bacteria, while others being specific for the eukaryotic 80S ribosome. We found that minor changes in the polypeptide-coding sequence can drastically alter the sensitivity of protein translation towards a particular macrolide. We also show that by altering the structure of the macrolide antibiotics bound to the eukaryotic ribosome, it is possible to modulate their effect upon synthesis of individual proteins.

## Results

### A single rRNA mutation sensitizes yeast cells and cytoplasmic 80S ribosomes towards extended macrolide antibiotics

**Fig. 2:**
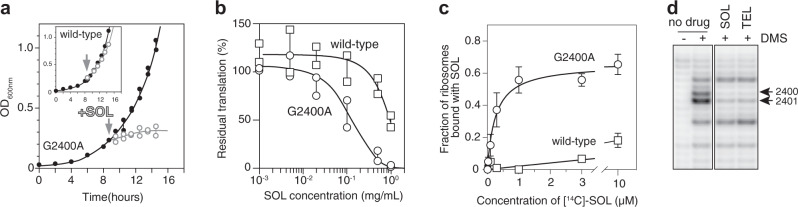
Macrolide antibiotics bind to the mutant yeast ribosome and inhibit protein synthesis and cell growth. **a** SOL arrests growth of the G2400A mutant *S. cerevisiae* cells. Plots show the growth of mutant cells in the absence of the drug (filled black circles) or after addition of 8× MIC of SOL (open gray circles). The inlet shows that wt yeast is resistant to SOL. The graphs represent results of two independent experiments with individual data points indicated. **b** Residual protein synthesis in wt (squares) and G2400A mutant (circles) yeast cells exposed to SOL. Translation was assessed by measuring incorporation of l-[^35^S]-methionine into polypeptides after exposure of cells for 10 min to different concentrations of SOL. l-[^35^S]-methionine incorporation in the untreated mutant cells was set to 100%. The data points of two independent experiments are indicated by squares (wt) and circles (G2400A mutant). **c** Equilibrium binding of [^14^C]-SOL to wt (squares) or G2400A mutant (circles) ribosomes. The data are presented as mean values of three independent experiments; error bars show standard deviation. **d** SOL and TEL protect A2400 and A2401 of the 25S rRNA from chemical modification. The gel shows the primer extension analysis the 25S rRNA extracted from the untreated or dimethyl sulfate (DMS)-modified G2400A 80S ribosomes incubated without or with the antibiotics. The position of the cDNA bands corresponding to the nucleotide residues A2400 and A2401 are indicated by the arrows. The chemical modification of wt ribosomes in the presence of SOL and TEL and that of mutant ribosomes in the presence of ERY and azithromycin are shown in Supplementary Fig. 2b, c, respectively. The uncropped gel can be found in the Source data file. The result is a typical representative of two independent experiments.

A previous work had shown that the 25S rRNA G2400A mutation (A2058 in *E. coli*; through the rest of the text, the *E. coli* numbering is shown in parentheses) in the macrolide binding site, which replaced the eukaryote-specific guanine with bacteria-specific adenine, did not render yeast 80S ribosomes sensitive to ERY^34^. We reasoned that newer and more active macrolides such as TEL or SOL (Fig. 1), with extended side chains that establish additional interactions in the NPET of the bacterial ribosome^15,35,36^, could perhaps bind to the G2400A mutant yeast ribosomes. Therefore, we engineered the G2400A mutation de novo in the *S. cerevisiae* strain NOY891, where the rRNA-encoding *RDN* locus on chromosome XII is deleted and rRNA is expressed from a plasmid^37,38^ (see “Methods” section). Primer extension analysis confirmed that the cytoplasmic 80S ribosomes in the resulting strain contained exclusively the mutant G2400A 25S rRNA (Supplementary Fig. 2a). In agreement with the previous report^34^, the engineered G2400A mutant yeast remained resistant to ERY and the closely related azithromycin (AZI). However, TEL and SOL completely abolished the growth of the mutant at concentrations below 200 µg/ml (TEL) or below 50 µg/ml (SOL) (Fig. 2a and Supplementary Table 1). The growth arrest was likely caused by inhibition of protein synthesis because exposure of the mutant yeast cells to SOL resulted in a rapid decline of incorporation of l-[^35^S]-methionine into newly synthesized polypeptides (Fig. 2b). Thus, the G2400A mutation in 25S rRNA of the large subunit of the eukaryotic cytoplasmic ribosome sensitized yeast to the extended macrolide antibiotics. We used the engineered mutant to explore the mode of binding of this class of inhibitors to the 80S ribosome, and the effects of macrolides upon translation in the eukaryotic cell.

### Extended macrolides bind in the exit tunnel of the mutant yeast ribosome

To verify that extended macrolides inhibit protein synthesis in the mutant yeast cells by acting upon ribosomes, we carried out binding studies using radiolabeled [^14^C]-SOL. While wild-type (wt) ribosomes were essentially impervious to binding of SOL, ribosomes with the G2400A mutation showed a significantly increased affinity to the drug, allowing binding with an apparent dissociation constant in the sub-micromolar range (*K*_d_ = 0.26 µM ± 0.09) (Fig. 2c). To further analyze whether binding of SOL and TEL takes place in the NPET of the mutant eukaryotic ribosome, we analyzed drug-rRNA interactions by chemical RNA probing. In bacteria, NPET-bound macrolides shield A2058 and A2059 in the 23S rRNA from modification by dimethylsulfate (DMS)^39–41^. In mutant (but not wt) yeast ribosomes, SOL and TEL protected the two equivalent rRNA residues, A2400 and A2401, from DMS modification (Fig. 2d and Supplementary Fig. 2b) revealing binding of these antibiotics in the NPET of the 80S ribosome. ERY and AZI that lacked the alkyl-aryl side chain present in the extended macrolides afforded only marginal protection of these residues (Supplementary Fig. 2c), corroborating a very weak binding of these antibiotics, which therefore, were excluded from the subsequent experiments.

**Fig. 3:**
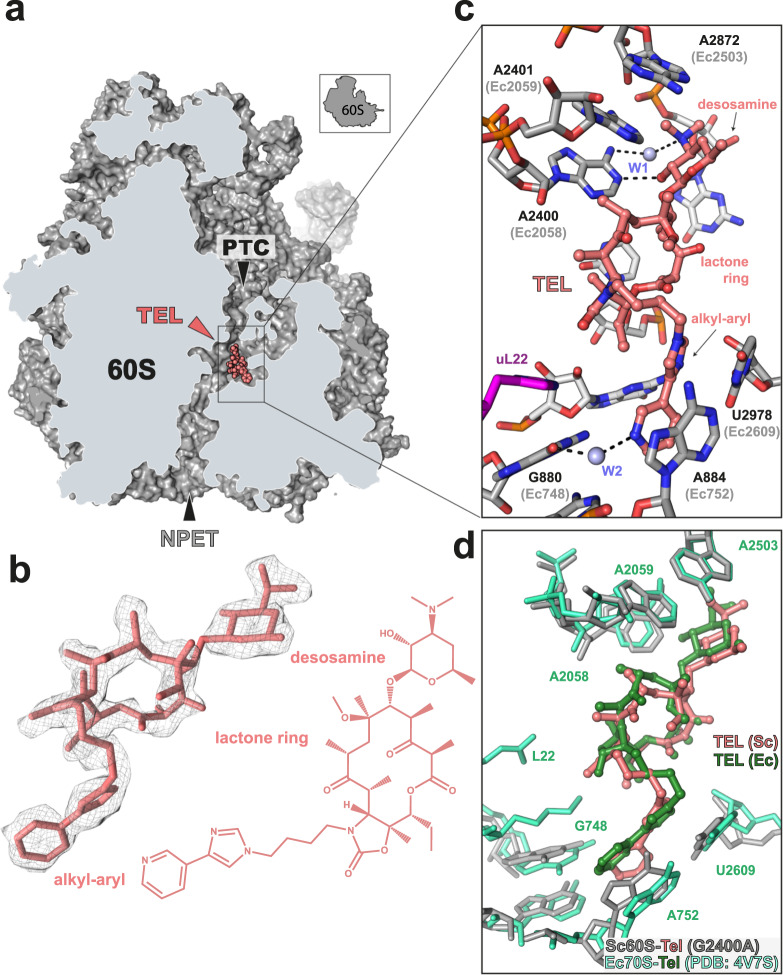
Cryo-EM structure of TEL bound to the yeast ribosome. **a** Transverse section of the cryo-EM map density (gray) of the large (60S) subunit of the yeast G2400A mutant ribosome with TEL (salmon) bound within the NPET. **b** Isolated cryo-EM density for TEL (gray mesh) with fitted molecular model for TEL. The similarly-oriented chemical structure of TEL is shown for reference. **c** TEL bound within the NPET with the surrounding nucleotides of the 25S rRNA (gray) and His133 residue of uL22 protein (purple). The N1 of A2400(A2058) forms a hydrogen bond interaction to the hydroxyl group of the desosamine sugar of TEL and a water molecule (W1) mediates interaction of the desosamine’s dimethylamine with the N6 of A2400(A2058), as was also observed in bacteria^43^. The alkyl–aryl sidechain of TEL stacks upon the base pair A884(A752)-U2978(U2609) and forms bridging interaction with water W2 (light blue) and O6 of G880(G748). **d** Superposition of TEL bound to the *E. coli* ribosome (green, PDB ID 4V7S^15^) with TEL (salmon) in complex with the *S. cerevisiae* 60S subunit (Sc60S) bearing the G2400A mutation. *E. coli* rRNA and ribosomal protein residues are light green, yeast rRNA and protein residues are gray.

While revealing the general location of the macrolide binding site in the mutant yeast ribosome, the results of rRNA probing are unable to reveal the atomic interactions and the exact orientation of the drugs in the 80S ribosome NPET, which can be dramatically affected by the distinct architectures of ribosomes from different species^42^ (Supplementary Fig. 1). To understand the precise binding mode of the extended macrolides in the yeast ribosome, we determined the cryo-EM structure of the mutant G2400A ribosome in complex with TEL with an average resolution of 3.1 Å (Supplementary Fig. 3). The resolution was further improved with focused refinement on the large 60S subunit (Supplementary Fig. 3), leading to a final reconstruction of the yeast 60S-TEL complex (Fig. 3a) with an average resolution of 2.9 Å and extending to 2.5 Å within the core of the particle (Supplementary Fig. 4a–d and Supplementary Table 2). The density for TEL observed within the NPET of the 60S subunit was well-resolved, enabling an unambiguous placement of the 14-membered macrolactone ring, C5-desosamine sugar and the C10–C11 alkyl–aryl side chain (Fig. 3b and Supplementary Fig. 4e). TEL binds within the NPET of the yeast large ribosomal subunit with desosamine sugar extending towards the PTC and placed adjacent to 25S rRNA nucleotides A2400 (2058) and A2401 (2059) (Fig. 3c), consistent with the DMS protection experiments (Fig. 2d). The hydroxyl group of the desosamine forms a hydrogen bond interaction with the N1 of A2400, whereas the dimethylamino group appears to interact with the exocyclic N6 amine group of A2400 via a water molecule (W1) (Fig. 3c and Supplementary Fig. 4f), as reported previously for ERY^43^. The alkyl–aryl side chain of TEL is stretched in the opposite direction with its aromatic moiety stacked upon the base pair formed by A884 (752) and U2978 (2609) (Fig. 3c and Supplementary Fig. 4g), similar to that observed for TEL binding to *E. coli* (Fig. 3d), *T. thermophilus* and *B. subtilis* ribosomes^15,16,44^, and distinct from its placement in the ribosomes of the bacterium *Deinococcus radiodurans* (Berisio, 2003 #4141;Schlunzen, 2003 #3935) and the archaeon *Haloarcula marismortui* (Tu, 2005 #4874) (Supplementary Fig. 5a, b). We also detect additional density that would be consistent with a water molecule (W2) forming a bridging interaction between the side chain of TEL and the O6 of G880 (748) of the 25S rRNA (Fig. 3c and Supplementary Fig. 4g), and helping to orient the alkyl-aryl appendage for its interaction with the A884 (752)—U2978 (2609) base pair. Curiously, in the yeast 60S-TEL structure, the aromatic rings of the alkyl-aryl side chain are non-planar and rotate with respect to each other, thereby enabling an optimal stacking interaction with the nucleobases of A884 and U2978—which are themselves non-planar (Fig. 3c). In all previous structures of TEL complexed with bacterial or archaeal ribosomes^14–16,36,44,45^, the alkyl-aryl moiety has been modeled in a planar conformation, regardless of whether the A884-U2978 base-pair is planar or not (Fig. 3d and Supplementary Fig. 6a, b). It remains unclear whether these distinctions are due to species-specific differences or the limited resolution in some of the previous structures. Comparison of the yeast 60S-TEL complex determined here with vacant yeast 80S ribosomes^46^, did not reveal any significant conformational change within the 25S rRNA nucleotides that comprise the TEL binding site (Supplementary Fig. 5c). Thus, it is TEL that adjusts its conformation to establish optimal interactions with the ribosomal site, rather than requiring reorientation of the ribosomal residues for the optimal fit. Mutating in silico A2400 back to G in our structure or aligning the TEL-60S complex with the vacant wt yeast 80S ribosomes^46^, suggests that the N2 position of G2400 would mildly clash with the desosamine sugar of TEL (Supplementary Fig. 7). In addition, the presence of wt G2400 would preclude establishing the water (W1) mediated interaction of the dimethylamino group of TEL with this 25S rRNA residue (Supplementary Fig. 7b), as observed for ERY on bacterial ribosomes^43^. Collectively, these observations provide a rationale for the reduced affinity of TEL to wt yeast ribosomes (Supplementary Fig. 2b), and pave the way for rational design of macrolides that could be active against wt eukaryotic ribosomes.

### Macrolides elicit context-specific ribosome stalling in yeast cells

**Fig. 4:**
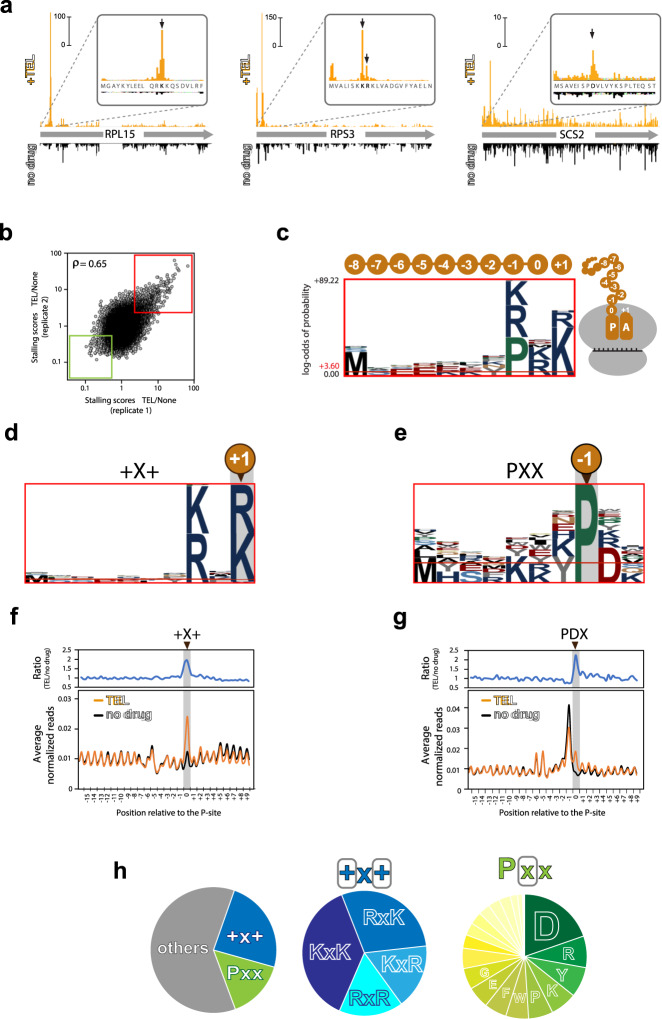
Macrolides arrest translating ribosomes at specific sequences. **a** Examples of TEL-induced translation arrest within *S. cerevisiae* genes. Comparison of ribosome footprint densities in untreated G2400A yeast cells (“no drug”, downwards extending black plots) or cells treated for 10 min with 1.5 mg/mL (8× MIC) of TEL (upwards-extending orange plots). The arrows indicate prominent footprint density peaks; the amino acid in the P site of the stalled ribosome is shown in bold. **b** The correlation of pause score ratios in TEL-treated and untreated cells for 46,445 sites within the actively expressed genes between two independent experiments. The red rectangle includes the 749 sites with ≥2.5-fold enrichment of ribosomal density in TEL samples in both experiments, which were used for the pLogo analysis shown in **c**–**e**. The green rectangle marks the 1809 sites of the least efficient TEL action (pause scores reduced by ≥2-fold in the TEL-treated samples). **c** pLogo analysis of the sequences of the nine C-terminal residues of the nascent protein (positions 0 to −8) and the A-site amino acid (position +1) at the sites of strongest TEL action. **d** pLogo analysis of the sites of the strongest TEL action conforming to the +X+ arrest motif. **e** pLogo analysis of the strongest arrest sites where proline (P) is present in the penultimate position of the nascent chain. **f**, **g** Bottom panels: Metagene analyses of ribosome density around the +X+ (**f**) and PDX (**g**) arrest motifs in TEL-treated (orange plots) and untreated (black plots) cells. Top panels show the ratio between the TEL and control metagene plots. The P-site codons of the +X+ and PDX motifs (highlighted by gray shadowing) are assigned as position 0. Note that the large metagene peak upstream from the highlighted peak in the PDX plot results from TEL-independent ribosome stalling at Pro codons^54^. **h** Relative occurrence of the different prevalent motifs among the 749 sites of strongest TEL-induced arrest. Left pie-chart shows all 749 sites, middle chart represents the subset of sites conforming to the +X+ motif and the chart on the right illustrates the subset of sites corresponding to the PXX motif.

Having demonstrated that macrolides can interfere with translation by binding in the NPET of the mutant eukaryotic ribosome, we wanted to know whether, similar to their action in bacteria^10,20,21^, they preferentially arrest the ribosome at specific sequence motifs. To address this question in an unbiased way, we analyzed re-distribution of ribosomes on the mRNAs in the TEL-exposed *S. cerevisiae* cells by ribosome profiling (Ribo-seq)^47^. In order to minimize secondary stress-related effects, the exponentially growing G2400A yeast cells were exposed for only a brief time (10 min) to a high concentration of TEL (1.5 mg/mL equivalent to 8× MIC), that result in ~90% inhibition of protein synthesis (Supplementary Fig. 8a, b). Under these conditions, the polysome profile of the treated cells showed virtually no changes in comparison with the untreated control (Supplementary Fig. 8c). The Ribo-seq data were highly reproducible between the biological replicates, when gene scores (the relative densities of ribosome footprints mapped to a gene) or pause scores (the relative codon occupancies) were compared (Supplementary Fig. 8d–f). However, comparison of TEL datasets relative to the controls showed a number of sites with a notably increased pause score in the antibiotic-treated samples, reflecting a redistribution of ribosomes within mRNAs in response to the exposure to the drug (Supplementary Fig. 8g–i). This could be clearly observed in individual ORFs, where exposure to TEL caused a significant increase in the ribosomal occupancy of specific codons (Fig. 4a), revealing drug-induced stalling of translation at defined sites within coding sequences. At the same time, the distribution of ribosomes within some other ORFs was barely affected by the TEL treatment, suggesting that macrolides affect eukaryotic translation in context-selective and protein-selective manner.

To determine whether specific sequence signatures are associated with the sites of most prominent macrolide-induced translation stalling, we applied pLogo analysis^48^ to 749 sites where the relative ribosome occupancy (pause score) was ≥2.5-fold higher in the TEL-treated cells compared to the control in two independent biological replicates (Fig. 4b, upper right quadrant). According to the location of the TEL binding site in the NPET of the yeast ribosome (Fig. 3), the drug could make contact with C-terminal residues of the nascent polypeptide^6,49^. Therefore, we looked for a possible enrichment of specific amino acids within the nine C-terminal residues of the nascent chains (residues −1 to −8 and residue number 0 assigned to the P-site amino acid) and, in addition, the incoming amino acid (position +1, encoded by the codon located in the A site of the arrested ribosome). There was a strong tendency for the prevalence of Pro, Lys, or Arg as the penultimate residues of the nascent chain, and of Lys (and to a lesser extent of Arg) as the incoming amino acid (Fig. 4c). Fixing defined amino acid residues at a specified position of the pLogo plot allowed the identification of the two main TEL arrest motifs in yeast (Fig. 4d–h). The most common motif (24% of the strong arrest sites) is represented by the sequence Arg/Lys-X-Arg/Lys (where X, which could be any amino acid, corresponds to the C-terminal residue of the nascent peptide in the stalled ribosome) (Fig. 4d). The genome-wide metagene analysis confirmed that TEL treatment leads to a significant enrichment of ribosome footprints at the codon specifying the middle amino acid of the Arg/Lys-X-Arg/Lys motif (Fig. 4f, h). This pattern matches the one commonly found in TEL-arrest sites in bacteria^20,21^ called the “+X+” motif^11,21^ because both Arg and Lys side chains carry a positive charge. The second most prevalent motif, found in 15% of the strongest arrest sites in yeast, is characterized by the presence of proline as the penultimate amino acid of the nascent protein (Fig. 4e, h). Because among such sites the most frequent C-terminal residue is an aspartate (Fig. 4h), we designated this motif as PDX. Genome-wide metagene analysis confirmed the general TEL-induced ribosome stalling at the middle codon of the PDX arrest motif (Fig. 4g).

Noteworthy, although the PDK sequence is one of the preferred sites of TEL-induced arrest in bacteria^21^, the general PDX sequence is not particularly problematic for the TEL-bound bacterial ribosome^11,21^. While the variable X amino acid within the +X+ and PDX motifs might play a secondary role, its identity apparently modulates the efficiency of stalling (Supplementary Fig. 9a, b). Importantly, not every +X+ or PDX sequence, even when containing a favorable X amino acid within the motif, was associated with increased ribosome occupancy in the TEL-treated yeast cells (Supplementary Fig. 9c). Therefore, the general signal of macrolide-induced stalling likely includes additional elements besides the identified short motifs. No well-defined sequence motifs were detected for the remaining 61% of the strongest sites of TEL-induced arrest that do not conform to the +X+ or PDX motifs (Fig. 4h), except possibly for an increased incidence of the presence of Lys and Arg in the P-site of the TEL-stalled ribosome (Supplementary Fig. 9d).

We also analyzed the mRNA sites, where translation was least sensitive to inhibition by TEL. Due to drug-induced redistribution of ribosomes on mRNAs, such codons are characterized by diminished pause scores in the TEL-treated cells relative to the control (Fig. 4b, lower left quadrant). The yeast ribosome is least susceptible to TEL inhibition when the nascent chain contains Asn at the C-terminus, or when Glu is the incoming amino acid (Supplementary Fig. 9e, f). However, we did not find these two residues to be associated in a single sequence context. Rather, their effect on reducing TEL inhibition appeared to be independent from each other (Supplementary Fig. 9f). Neither a C-terminal Asn within the +X+ stalling motif, nor a Glu residue in the PDX arrest sequence are able to counteract the TEL action (Supplementary Fig. 9a, b), indicating that their effects are fairly moderate in comparison with the impact of the arrest motifs on translation when the drug is present.

Altogether, the Ribo-seq analysis revealed that in the eukaryotic cell macrolides act as context specific inhibitors, arresting translation preferentially at specific sequence motifs.

### Extent of inhibition of protein expression by TEL depends on the polypeptide sequence

Although Ribo-seq analysis showed that TEL-bound yeast ribosomes stall at distinct sites during translation of a gene, the redistribution of ribosome footprints on mRNA does not directly reveal how the yield of the encoded protein is affected. To explore whether the context specificity of macrolide action can be manifested as sequence-selective inhibition of protein expression, we studied the effects of TEL on the synthesis of individual proteins in a cell-free system driven by the G2400A yeast ribosomes.

**Fig. 5:**
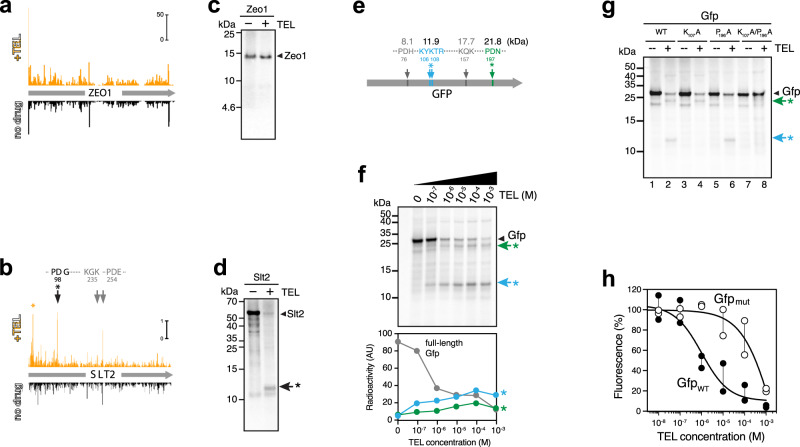
TEL selectively inhibits in vitro translation of proteins with specific sequence motifs. **a, b** Ribosome footprint density in the *ZEO1* (**a**) and *SLT2* (**b**) genes in yeast cells treated (orange plots) or not (black plots) for 10 min with 8× MIC of TEL. PDX and +X+ motifs present in the *SLT2* gene are indicated by arrows. Increased ribosome occupancy at early codons of *SLT2* (orange asterisk) occurs at sites not conforming to the +X+ or PDX motifs. **c, d** SDS-gel analysis of the [^35^S]-radiolabeled proteins generated by in vitro translation of the *ZEO1* (**c**) or *SLT2* (**d**) templates by the G2400A yeast ribosome in the absence of the drug or in the presence of 100 µM TEL. Arrowheads indicate full-size proteins. The truncated Slt2 polypeptide, likely a product of TEL-mediated translation arrest at the PD_98_G motif, is indicated by an arrow and asterisk. **e** Locations of +X+ and PDX motifs in the *GFP* reporter gene. The molecular weights (kDa) of the proteins whose translation would be terminated at the corresponding sites are indicated. **f** Top: SDS-gel analysis of the [^35^S]-labeled products of in vitro translation of the *GFP* template in the absence or presence of increasing concentration of TEL. The band of the full-size Gfp is indicated. Truncated Gfp polypeptides resulting from TEL-induced ribosome arrest are marked with arrows and asterisks (colored as shown in **e**. Bottom: quantification of the relative radioactivity associated with the full-size and truncated Gfp polypeptides in the SDS gel above. **g** SDS-gel analysis of the in vitro translation products of the wild-type (WT) *GFP* gene or its mutant variants in the absence or presence of 100 µM TEL. Translation products are labeled as in **f**. **h** Expression of wt Gfp (Gfp_wt_) or its K_107_A/P_196_A mutant (Gfp_mut_) in the yeast cell-free translation system in the presence of increasing concentrations of TEL. Accumulation of functional Gfp was followed by its fluorescence. The activity of Gfp_wt_ in the “no-drug” samples was set at 100%. The curves represent average of two independent experiments with individual data points indicated by filled (Gfp_wt_) or open (Gfp_mut_) circles. The uncropped gel can be found in the Source data file. The results shown in **c**, **d**, **f**, and **g** are typical representatives of at least two independent experiments.

Cell-free expression of the Zeo1 protein, encoded by an ORF where the ribosome footprints pattern remained unchanged in the TEL treated cells, was barely affected even by high concentrations of the macrolide (Fig. 5a, c). By contrast, expression of the Slt2 polypeptide was highly sensitive to TEL and instead of the full-size protein, a shorter (~11 kDa) product accumulated (Fig. 5b, d). Appearance of the truncated Slt2 fragment possibly results from translation arrest at the PDG_98_ sequence of the *SLT2* ORF (Fig. 5b, d) that conforms to the PDX stalling motif.

To directly test whether the protein sequence impacts the extent of inhibition of its translation by TEL, we followed expression of the reporter, superfolder Gfp (Gfp), in a cell-free system^50^. Five potential macrolide arrest motifs could be recognized within the Gfp sequence, two of the PDX type (PDH_77_ and PDN_198_) and three of the +X+ type, two of them in tandem, KYKTR_109_, and the single KQK_158_ (Fig. 5e). Consistently, in vitro synthesis of the full-length (26.7 kDa) Gfp was inhibited by TEL in a concentration dependent manner (Fig. 5f). Two distinct truncated polypeptides with apparent molecular weights of ~12 kDa and ~22 kDa appeared, when the Gfp template was expressed in the cell-free system in the presence of TEL (Fig. 5f, g, green and blue arrows and asterisks). The smaller product may originate from interruption of *GFP* translation at the KYKTR_109_ site, whereas the accumulation of the 22 kDa product could be caused by translation arrest at the PDN_198_ sequence. To test these assertions, we introduced mutations disrupting the predicted arrest sequences. Replacing the Lys_107_ codon with an Ala codon (K_107_A mutation), converted the encoded KYK_107_TR sequence to KYA_107_TR and thereby eliminated both overlapping +X+ motifs. When this mutant template was translated in the presence of TEL, the 12 kDa peptide no longer appeared (Fig. 5g, compare lanes 2 and 4), confirming that the wt KYKTR_109_ sequence was the site of the drug-induced translation arrest. Similarly, inactivation of the PDX motif by mutating the Pro_196_ codon within the PD_197_N sequence to the Ala codon (P_196_A mutation) abolished the appearance of the 22 kDa product (Fig. 5g, compare lanes 2 and 6). The double K_107_A/P_196_A mutant that lacked both of the aforementioned macrolide arrest sites in *GFP*, prevented accumulation of both the 12 and 22 kDa truncated polypeptides and rendered translation of the full-size protein highly resistant to inhibition by TEL (Fig. 5g, compare lanes 2 and 8). In an independent assay, where Gfp expression was monitored by following its fluorescence, even at concentrations of TEL as high as 100 µM, translation of the full-size protein carrying the K_107_A/P_196_A mutations remained at ~75% of the control, whereas production of wt Gfp was reduced to a lesser than 20% level (Fig. 5h). These results demonstrate that inhibition of protein production by binding of a macrolide to a eukaryotic ribosome critically depends on the sequence of the translated polypeptide, and revealed macrolides as potential protein-selective inhibitors of eukaryotic translation.

### Context-specific inhibition of translation depends on the structure of the macrolide antibiotic

**Fig. 6:**
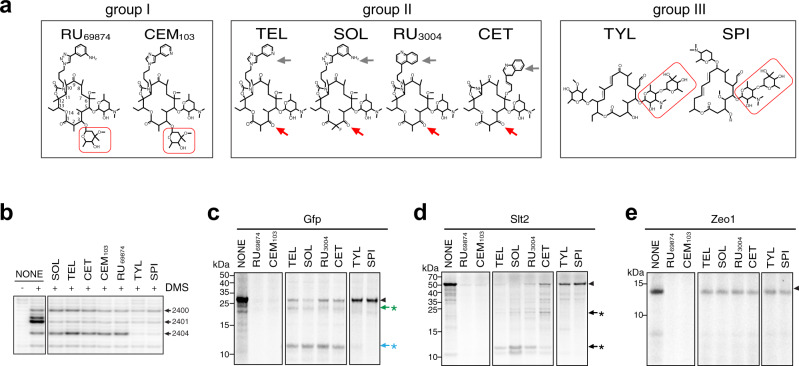
The structure of the macrolide affects selective inhibition of translation. **a** Structures of representative macrolide antibiotics grouped according to their chemical features: group I, 14-atom macrolactone ring carrying C3 cladinose sugar (framed in red); group II, 14-atom macrolactone ring with C3 keto group (red arrow) and extended alkyl-aryl side chain (gray arrow); group III, 16-member macrolactone with C5 disaccharide chain (framed in red). **b** Chemically different macrolides readily bind to the yeast G2400A mutant ribosome as revealed by the protection of 25S rRNA residues A2400(2058), A2401(2059) and, in the case of the group III compounds, A2404(2602) from DMS modification. The drugs were present at 100 µM. The primer extension products were resolved in the denaturing 6% polyacrylamide gel. Control samples (“NONE”) contained no macrolides. **c**–**e** SDS-gel analysis of the [^35^S]-labeled protein products accumulated during translation in the yeast cell-free system of templates encoding the Gfp reporter (**c**) or the Slt2 (**d**), and Zeo1 (**e**) yeast proteins. Translation reactions were supplemented with 100 µM of the indicated macrolides. The control reaction (NONE) contained no antibiotic. Arrowheads and arrows/asterisk represent the bands corresponding to the full-length and truncated products, respectively. The uncropped gel can be found in the Source data file. **b**–**e** Representative gels of two independent experiments.

We asked whether variation in the structure of the drug bound in the NPET of the yeast ribosome would affect specificity of translation inhibition. Several representative extended macrolides differing in the structure of the macrolactone ring and side chains were selected for these experiments (Fig. 6a). RNA chemical probing showed that in contrast to ERY or AZI, all these extended macrolides efficiently bind to the mutant yeast ribosome, and protect the A2400 and A2401 residues (A2058/A2059) from DMS modification (Fig. 6b and Supplementary Fig. 2c). Tylosin (TYL) and spiramycin (SPI), that carry a C5 disaccharide side chain (boxed in Fig. 6a), additionally protected A2404 (A2062) (Fig. 6b). Such protection, caused by a direct interaction of the drugs’ C5 side chain with this nucleotide, which has been noted previously in archaeal and bacterial ribosomes^51,52^, is indicative of a similar binding mode of these drugs in the eukaryotic ribosome.

We then examined the effects of all these antibiotics on the in vitro synthesis of three different polypeptides (the Gfp reporter and the two yeast proteins Slt2 and Zeo1), that we used in the previous experiments (Fig. 5). Strikingly, different drugs affected protein synthesis in a very distinct fashion. The C3-cladinose-containing compounds RU69874 and CEM103 efficiently blocked the expression of all three proteins, including TEL-resistant Zeo1 (Fig. 6c–e). Because no accumulation of truncated peptides was observed, these inhibitors possibly block protein synthesis at very early stages of cell-free translation. TEL, SOL, cethromycin (CET), and RU_3004_, all of which carry the C3-keto group instead of cladinose, had only a small effect on expression of Zeo1 (Fig. 6e), but readily inhibited translation of Gfp and Slt2 (Fig. 6c, d). As we had observed with TEL (Fig. 5d, f, g), SOL, CET, and RU_3004_ caused accumulation of specific truncated polypeptide products due to translation arrest at the identified macrolide arrest motifs within the Slt2-coding and Gfp-coding sequences. The remaining two drugs, 16-membered ring TYL and SPI with C5 disaccharide side chains barely affected expression of the tested proteins in the cell-free system (Fig. 6c–e) in spite of the robust binding of these compounds to the G2400A ribosome (Fig. 6b). The efficient synthesis of full-length polypeptides potentially reflected the ability of the yeast ribosomes to polymerize the problematic motifs regardless of the presence of these drugs. TYL and SPI also failed to inhibit growth of the mutant yeast cells (Supplementary Table 1).

Taken together, our data indicate that by altering the structure of macrolide antibiotics bound to the eukaryotic ribosome it is possible to modulate their effect upon synthesis of individual proteins.

## Discussion

In this study, we demonstrated that binding of macrolide antibiotics in the NPET of a mutant yeast 80S ribosome inhibits protein synthesis in a context-specific manner. Two major sequence motifs, +X+ and PDX, were identified as problematic for polymerization by the TEL-bound yeast ribosome. Elimination of the arrest sequences significantly improved the expression of an otherwise drug-sensitive protein in the presence of the antibiotic. These results establish macrolides as prospective protein-selective inhibitors of eukaryotic translation.

The main arrest motif in yeast, +X+, is identical to the primary motif of macrolide-induced stalling in bacteria^20–22,25^. The second motif, PDX (or more generally, PXX) appears to be more specific for the eukaryotic cytoplasmic ribosome. Although prolines are also found in some of the macrolide arrest motifs in bacteria, they are most commonly placed in the P or A sites of the stalled ribosome^11,20,21^. In contrast, in the TEL-bound yeast ribosome, the presence of proline in the penultimate position of the nascent protein creates a hurdle for translation. Importantly, neither in bacteria, nor in yeast, the nascent chain residues critical for the drug-induced ribosome stalling are juxtaposed with the drug molecule in the NPET. Instead, they are located at the PTC or in its immediate vicinity. Thus, in eukaryotes, just like in bacteria, TEL appears to act as an inhibitor of peptide bond formation between specific donor and acceptor substrates rather than as a discriminating gateway for the growing protein chain.

Interestingly, both macrolide arrest motifs, +X+ and PDX, were found previously at the sites of ribosome pausing in yeast cells depleted of the elongation factor eIF5A, which helps the ribosome in polymerizing problematic combinations of amino acids^53–55^. It is possible, therefore, that binding of TEL to the yeast ribosome aggravates the burden of polymerizing intrinsically-difficult sequences. Noteworthy, however, in the untreated cells the ribosome often pauses at proline codons, with the nascent chain ending with proline^54^ (Fig. 4g). TEL additionally pauses translation at the following codon, when a proline residue is present in the penultimate position of the nascent chain (Fig. 4g). The pLogo plots also showed a somewhat increased presence of methionine at position −8 relative to the site of TEL-induced arrest (Fig. 4c–e and Supplementary Fig. 9d). However, this effect may simply reflect the fact that in the ten amino acid window that we used in our analysis, the initiator Met will be always found at this position when the window is placed at the beginning of the ORFs.

The macrolide arrest motifs identified by our bioinformatics analysis likely represent only a part of the signal required for the drug-induced ribosome stalling. Indeed, the Ribo-seq data revealed that the TEL-bound ribosome stalls only at a fraction of the sequences matching the identified arrest motifs. This conclusion was additionally reinforced by our in vitro translation experiments, where only some of the +X+ or PDX sequences caused accumulation of the corresponding truncated polypeptides (Fig. 5f, g). These observations argue that other factors, operating at the level of the polypeptide chain, mRNA or tRNA might be also at play. In particular, more remote segments of the nascent protein, within or even outside of the NPET, could suppress or stimulate the arrest imposed by the tunnel-bound antibiotic, similar to the influence of the distal nascent chain context on ribosomes pausing during polymerization of polyproline sequences^56,57^ or of the native stalling peptides^58,59^.

The effect of macrolides on eukaryotic translation critically depends on the structure of the NPET-bound antibiotic (Fig. 6). The ketolides (macrolides with a C3-keto group) exhibit strong context dependence of the translation arrest (Fig. 6c–e). Similar compounds but with a C3 cladinose inhibit protein synthesis but do not yield any truncated protein products, likely because they interfere with the very early stages of translation (Fig. 5c–e). Strikingly, the 16-member macrolactone ring containing drugs TYL and SPI, in which a disaccharide moiety replaces the C5 desosamine present in other tested macrolides, had only limited effect on the yield of the three tested proteins (Fig. 6c–e). One possibility is that the advancing N-terminus of the growing polypeptide could displace TYL or SPI from their binding site, as has been suggested for the mode of action of short macrolide resistance peptides in bacteria^60–62^. Alternatively, these antibiotics could be much more selective compared to ketolides and the three proteins used in our in vitro experiments simply lacked the required arrest motifs. Noteworthy, some reports alluded that TYL could induce premature stop codon readthrough in mammals^63^, suggesting a possible effect of the antibiotic on translation in the wt eukaryotic cell, although it remains unknown whether this activity was mediated by binding of the drug to the ribosome.

Besides macrolides, the only other known highly-selective protein synthesis inhibitor acting upon the eukaryotic ribosome is the compound PF846, which interferes with translation of a very narrow subset of proteins in mammalian cells^5^. The binding site of PF846 in the NPET partially overlaps with that of macrolides (Supplementary Fig. 10), but its specificity and mode of action are significantly different. In contrast to TEL, which arrests translation at distinct sites, PF846 slows down the progression of the ribosome over several consecutive mRNA codons at the site of arrest^5–7^. At each of these codons the ribosome operates with a different combination of donor and acceptor ligands and therefore, in contrast to TEL, PF846 specificity is less dependent on the nature of the PTC substrates, but rather on the unusual trajectory of the nascent chain in the NPET^6,7^. Consistently, while macrolides inhibit peptide bond formation^24–27^, PF846 interferes with ribosome translocation and with translation termination^6,7^. It is remarkable that in spite of these differences, PF846 and TEL achieve context-specific inhibition of translation by binding to overlapping sites in the NPET. Interestingly, it has been recently shown that the compound tetracenomycin X binds to bacterial and eukaryotic ribosomes in a cavity of the NPET located on the wall opposite to the macrolide binding site and appears to act, at least during bacterial translation, in a sequence-specific manner^64^. Therefore, the PTC-proximal NPET segment emerges as the best target for the inhibitors, whose action depends on the sequence context of the growing polypeptide.

Identifying compounds capable of selectively suppressing expression of unwanted proteins, especially those that are viewed as “undruggable targets”, is an attractive strategy for the development of new medicines. For example, upregulation of expression of ribosomal proteins uL6 (gene RPL9), eL15 (RPL15), and eL39 (RPL39) is associated with increased tumor growth and metastasis in some cancers^65–67^. Our Ribo-seq analysis showed that some of the strongest sites of TEL-induced translation arrest in yeast are located within the ORFs encoding these proteins (Supplementary Fig. 11a, b), and these sequences are highly conserved in the corresponding human genes (Supplementary Fig. 11c). Thus, macrolide derivatives capable of selective inhibition of expression of these and other oncoproteins could inform the advancement of new cancer therapies.

In our study, we used several available macrolides which have been specifically selected or optimized by the pharmaceutical industry for their action upon the bacterial ribosome and the lack of the effects upon eukaryotic translation. Therefore, we were compelled to carry out our experiments with the yeast ribosome that was intentionally sensitized to macrolide action by introducing the G2400A substitution in the drug-binding site. This single-nucleotide mutation allowed for binding with considerable affinity of several macrolide antibiotics with extended side chains. Noteworthy, some macrolides (e.g., TYL or SPI) are capable of binding to the NPET of the archaeal ribosomes^51^, in spite of the eukaryote-like nature of their macrolide-binding site. These observations argue that a targeted drug optimization, especially if guided by high-resolution structural data, could yield macrolide-inspired compounds active against the unaltered eukaryotic cytoplasmic ribosome. The recent progress in synthetic chemistry of macrolides^68^ makes obtaining such compounds within a realistic reach.

## Methods

### Reagents and radiochemicals

All chemicals were from Thermo Fisher Scientific or Sigma-Aldrich. Radiochemicals were from PerkinElmer ([^35^S]-l-methionine and [^32^P]-γ-ATP), American Radiolabeled Chemicals ([^14^C] ERY) or Cempra Pharmaceuticals ([^14^C] SOL). Premixed media and media components for yeast and bacteria growth were from Difco. Macrolide antibiotics used in the study were purchased from Sigma-Aldrich or obtained from Aventis or Cempra Pharmaceuticals.

### Yeast strains and plasmids

*S. cerevisiae* strain NOY891^38^ and plasmid pJD694^69^ were kindly provided by Dr. Dinman (University of Maryland). *S. cerevisiae* NOY891 (*MATa ade2-1 ura3-1 leu2-3 his3-11 trp1 can1-100 rdn1ΔΔ::HIIS3*) carries the *TRP1*-selectable plasmid pNOY353, that contains the wild type *RDN* operon encoding 35S pre-rRNA under the control of *GAL7* promoter^38^. Plasmid pJD694 contains a *URA3* selectable marker and carries the 35S pre-rRNA operon under the control of the tetracycline (doxycycline) repressible TET promoter^69^.

The *S. cerevisiae* strain with the G2400A mutation in the 25S rRNA gene was prepared as follows. The G2400A mutation was introduced in plasmid pJD694 by overlap-extension PCR^70^, and the mutant plasmid was transformed into the NOY891 strain^71^. Transformants were selected by first plating cells on SC-Ura-Trp minimal media with 10 μg/mL of doxycycline (to select for the presence of plasmid pJD694 but repress expression of the mutant 25S rRNA), followed by selection on plates with complete YPD medium supplemented with 300 µg/mL of hygromycin B^72^.

### Antibiotic sensitivity testing

*S. cerevisiae* strains were grown with shaking at 30 °C in YPD medium supplemented with 100 µg/mL of ampicillin (Amp). Exponentially growing cells were diluted with fresh medium to the final culture density of *A*_600_ = 0.005 and placed in 96-well plates (100 µL of culture per well). After addition of increasing concentrations of macrolide antibiotics, plates were incubated for 18 h at 30 °C. The minimal inhibitory concentration (MIC) was determined as the lowest concentration of antibiotic that prevented visible cell growth.

### Inhibition of bulk protein synthesis in vivo

*S. cerevisiae* NOY891 G2400A mutant cells were grown exponentially at 30 °C in YPD medium supplemented with Amp. When the culture density reached *A*_600_ ~0.5, aliquots of 1.5 mL were transferred to 15 mL polypropylene tubes (Falcon). A macrolide antibiotic, TEL or SOL, was added to different final concentrations, which were ranging from 15 µg/mL (0.08x MIC) to 6 mg/mL (32x MIC) for TEL and from 1 µg/mL (0.02x MIC) to 1 mg/mL (20x MIC) for SOL. Control cultures were left without antibiotic. Cultures were incubated with shaking at 30 °C for 10 min and aliquots of 250 µL were withdrawn to estimate protein synthesis rate by metabolic labeling. For this, cells were collected by rapid centrifugation (1 min at 14,000 × *g*), washed twice with SD medium (8% Yeast Nitrogen Base, 2% glucose) containing the corresponding concentrations of macrolide antibiotics and resuspended in 30 µL of fresh SD medium supplemented with 40 µg/mL of each amino acid except methionine. Reactions were initiated by addition of 1 µL of 11 µCi/µL of [^35^S]-l-methionine (specific activity 1175 Ci/mmol). After 5 min incubation at 30 °C, 25 µL of the tubes’ contents were spotted onto ∅ 25 mm Whatman 3MM paper disks. Disks were immediately immersed into a beaker containing 500 mL of 5% trichloroacetic acid (TCA). Following collection of all samples, the content of the beaker with the disks was boiled for 5 min and TCA was discarded. The TCA wash procedure was repeated one more time. Disks were rinsed with acetone, dried, placed in vials with 5 mL of scintillation cocktail, and the amount of radioactivity retained was determined by scintillation counting. The time course of TEL-induced inhibition of bulk protein synthesis was carried out following essentially the same protocol except that the cultures were incubated with the antibiotic for 0, 5, 10, and 40 min before metabolic labeling was carried out.

### Purification of yeast 80S ribosomes

*S. cerevisiae* G2400A mutant 80S ribosomes were purified for the structural studies according to the published protocol^73^. The ribosomes for the drug binding assays were purified using the same protocol except that sucrose gradient fractionation was replaced with pelleting through a 30% sucrose cushion in a buffer containing 20 mM Hepes-KOH, pH 7.5, 120 mM KCl, 8.3 mM MgCl_2_, 2 mM DTT, 0.3 mM EDTA. Specifically, ribosomes collected from the lysate by sequential differential precipitation with PEG 20 K (4% and then 9% w/v) were resuspended in 15 mL of buffer A (30 mM Hepes-KOH, pH 7.5, 150 mM KCl, 10 mM MgCl_2_, 8.5% mannitol, 2 mM DTT, 0.5 mM EDTA), layered over a 15 mL sucrose cushion, and centrifuged for 16 h in a Ti-70 rotor (Beckman) at 36,000 rpm (130,000×*g*) at 4 °C. The ribosome pellets were resuspended in a storage buffer containing 10 mM Hepes-KOH, pH 7.5, 50 mM KOAc, 10 mM NH_4_Cl, 2 mM DTT, 5 mM Mg(OAc)_2_, flash frozen in liquid nitrogen and stored at −80 °C.

### Analysis of binding of macrolides to yeast ribosomes

Purified ribosomes were diluted to 80 nM (*A*_260_ = 4) and combined with varying concentrations of [^14^C]-SOL (specific activity 53 Ci/mol) in 100 µL of binding buffer (80 mM Hepes-KOH, pH 7.5, 140 mM KCl, 1.5 mM DTT, 5 mM MgCl_2_). The reactions were incubated for 1 h at 30 °C. Following incubation, ribosomes were captured using 0.5 mg diethyl amino ethyl (DEAE) magnetic beads (BioClone). Beads were rapidly washed three times with 1 mL of ice-cold binding buffer and then resuspended in 100 µL of 1% SDS as described^74^. Ribosome-associated radioactivity was determined by scintillation counting. Data were analyzed using Prism software (GraphPad).

Analysis of binding of unlabeled drugs to ribosomes was performed by RNA chemical probing following the conventional procedure^41^, but with minor modifications. Briefly, ribosomes (final concentration 0.2 µM) and antibiotic (final concentration 100 µM) were incubated in 50 µL of binding buffer for 1 h at 30 °C. Two microliters of dimethylsulfate (DMS) diluted 1:5 in ethanol were added to the reactions and incubation continued for additional 10 min at 30 °C. The reactions were quenched by addition of 50 µL of stop solution containing 0.6 M NaOAc and 1 M β-mercaptoethanol. Ribosomes were ethanol-precipitated and rRNA was extracted. The extent of modifications of 25S rRNA residues in the macrolide binding site was assessed by primer extension using the 5′-[^32^P]-labeled primer 25S-2430 (Supplementary Table 3).

### Cryo-EM and single-particle reconstruction of 60S-TEL complex

The 80S-TEL complex was generated by incubating purified ribosomes with 50 µM TEL for 15 min on ice in buffer 10 mM Hepes-KOH, pH 7.5, 50 mM KOAc, 10 mM NH_4_OAc, 2 mM DTT, 5 mM Mg(OAc)_2_. Four microliters of the reaction solution (absorbance *A*_260_ = 5) were applied to pre-coated Quantifoil holey carbon supported grids (R3/3, 3 nm C, Cu 300 mesh, Q44689, C3-C18nCu30-01) and vitrified using a Vitrobot Mark IV (FEI). Data collection was performed on Titan Krios 300 kV TEM equipped with a K2 direct detection camera (Gatan). Images of single ribosome particles were aligned using MotionCor2^75^ and 329,333 particles were picked automatically using Gautomatch (https://www.mrc-lmb.cam.ac.uk/kzhang/) with an 80S ribosome (PDB ID 6S47^76^) as a reference and using default settings. Defocus values were determined using Gctf software^77^ (https://www.mrc-lmb.cam.ac.uk/kzhang/). Images were processed with Relion 3.0^78^. Picked particles were sorted by 2D classification and 242,959 ribosome-like particles were selected for initial 3D refinement using an *S. cerevisiae* 80S reference structure (PDB ID 6S47^76^) (Supplementary Fig. 3a). 3D classification yielded five classes with one class containing 80S ribosomes with only E-site tRNA (75,303 particles), three classes with 80S ribosomes and sub-stoichiometric E-site tRNA (combined 153,893 particles) and one lower resolution class with sub-stoichiometric E-site tRNA (13,763) (Supplementary Fig. 3b–d). Particles of combined classes 2–4 were 3D refined and resolution optimized by CTF refinement through Relion 3.0 resulting in an average resolution of 3.1 Å (unmasked) and 2.9 Å (masked) determined using the “gold-standard” criterion (FSC_0.143_) (Supplementary Figs. 3e and  4a, left). Final 3D reconstructions were corrected for the modulation transfer function and sharpened by applying a negative B factor estimated by Relion 3.0. ResMap^79^ was used for local resolution estimations.

### Molecular modeling of 60S-TEL complex

The molecular model of the 60S ribosomal subunit, containing ribosomal proteins and rRNA, were based on *S. cerevisiae* 80S ribosome (PDB ID 6Q8Y^46^) and the molecular model of TEL was based on *E. coli* 70S-TEL (PDB ID 4V7S^15^). Models were rigid body fitted into the electron density map using Chimera^80^. The models were manually adjusted and refined using Coot^81^, while regions with poor density were not manually adjusted based on the initial model. The final model was refined using Phenix^82^ with structural restraints calculated by Phenix eLBOW^83^. Model validation was carried out using Phenix and MolProbity Server^84^ (http://molprobity.biochem.duke.edu/). The map vs. model cross correlation at FSC_0.5_ was calculated by Phenix (1.19.2-4158^82^) comprehensive cryo-EM validation^85^ for each map individually using the final molecular model (Supplementary Fig. 4a, right). The statistics of the final model are presented in Supplementary Table 2.

### Preparation of figures with Cryo-EM structures

Figures were generated using PyMOL (Schrödinger, LLC) and structural superpositions were generated by alignment to the bacterial or mammalian large ribosomal subunits. Isolated densities and density images were created using Chimera^80^ and visualized using ChimeraX^86^.

The structures of the NPET surfaces (Supplementary Fig. 1c) were generated using the described algorithm^87^ with the following parameters: 60 Å cubic grid with adjacent grid points separated by 1 Å; 10 Å sphere radius for creating an outer shell of the large ribosomal subunit; 3 Å sphere radius for “filling-up” the internal cavities of the ribosome, including NPET. The image was generated using PyMOL (Schrödinger, LLC).

### Preparation of samples for Ribo-seq

Two independent experiments (carried out on different days and employing different library preparation protocols) were carried out for collecting the Ribo-seq data. *S. cerevisiae* G2400A mutant cells were grown exponentially at 30 °C in two 1 L flasks each containing 200 mL of YPD medium. When the culture density reached *A*_600_ ~ 0.6, TEL was added to the final concentration of 1.5 mg/mL (8x MIC) to one of the flasks. Cultures were incubated with shaking for 10 min and cells were collected by rapid filtration through Express Plus® Membrane filter (Millipore) as described^88^. Cells were rapidly frozen in liquid nitrogen and lysed using Mixer Mill MM400 (Retsch) with 300 µL of lysis buffer (10 mM Hepes-KOH, pH 7.5, 50 mM KOAc, 10 mM NH_4_Cl, 2 mM DTT, 5 mM Mg(OAc)_2_) without addition of cycloheximide. Lysates were cleared by centrifugation (10 min 20,000 × *g* at 4 °C) and supernatants were treated with 30 U/A_260_ of RNaseI (Ambion) at 4 °C for 5 min. Three hundred microliter of digested lysates were loaded onto 500 µL of 25% (w/v) sucrose cushion in buffer 20 mM Tris-HCl, pH 8.0, 140 mM KCl, 10 mM MgCl_2_ supplemented with 1× Complete protease inhibitor. Ribosomes were pelleted by centrifugation in a TLA100.2 rotor at 90,000 rpm (350,000 g), 4 °C for 1 h. Pellets were resuspended in 1% SDS and ribosome-protected mRNA fragments were isolated by phenol-chloroform extraction followed by electrophoresis in 15% denaturing gel. RNA footprints ranging in size between ~20 and ~35 nt were excised from the gel, eluted and converted to sequencing libraries as described by Becker et al.^89^ (for replicate 1) or McGlincy & Ingolia^88^ (for replicate 2). The libraries were sequenced at the NUSeq Core (Northwestern University) on Illumina HiSeq 4000 platform.

### Computational processing of ribosome profiling data

Ribosome profiling data were processed following the described algorithms^88,90^. Briefly, the fastq files were first trimmed to remove linkers and, when necessary, demultiplexed to obtain individual samples from pooled data using CUTADAPT^91^. To remove tRNA and rRNA reads, the files were then aligned to an index of noncoding RNAs with BOWTIE (version 1.1.2)^92^ using the following parameters: -v 2 -y. The fastq files after noncoding RNA removal step were then aligned to coding regions and splice junctions using BOWTIE (version 1.1.2)^92^ with the following parameters: -v 2 -y -a -m 1—best—strata (two mismatches allowed and multiple alignments suppressed), and using the R64-1-1 S288C reference genome assembly (SacCer3, Saccharomyces Genome Database Project). Only 25–34 nt footprints were included in the analysis. 3′ alignments of the footprints were generated and used for subsequent analysis. Reads per million (rpm) was computed by normalizing the read count at each nucleotide position by the total number of mapped reads and then multiplying that value by 10^6^.

### Metagene analysis

Metagene plots were constructed by calculating the average (normalized by the total rpm in a window around the site of interest of the 100 nt centered at the middle codon of the motif. Genes with features smaller than the window size were excluded.

### Calculation of pause scores

Pause scores were generated by dividing the rpm associated with each nucleotide by the average rpm of the gene. TEL-induced pauses were calculated by dividing the pause scores obtained from TEL-treated cells by the pause scores in untreated cells. Nucleotides with at least 0.5 were included in the analysis. The change in pause scores resulting from TEL treatment was calculated by computing the average pause score per codon, and then dividing codon pause score in the TEL sample by the codon pause score in the untreated sample.

Gene scores were calculated by adding together the total rpm mapping to each coding sequence. Reads were shifted by 18 nt from their 3′ ends to map the P-site. Total number of reads for each gene were normalized by length of the gene (by dividing by the length in kilobases) to obtain rpkm values (gene scores).

### Analysis of the amino acid context enrichment at the sites of TEL-induced ribosome stalling

The 10-amino acid long sequences associated with the sites of the most pronounced TEL-induced translation arrest were computationally extracted. Each sequence included amino acids encoded by the eight codons preceding the codon positioned in the ribosomal P site, and by the P-site and A-site codons. The sequences associated with the sites showing ≥2.5-fold change in pause score in both TEL samples compared to the control were compared to the amino acid sequences associated with all the codons (46,445 in the 8× MIC sample) included in the analysis using the pLogo tool^48^.

### Preparation of yeast lysate and in vitro translation

The yeast lysate for in vitro translation was prepared according to a procedure described by Hodgman *et al*.^93^ with minor modifications. The *S. cerevisiae* NOY891 G2400A mutant cells were grown at 30 °C in 1 L of YPD medium supplemented with 100 µg/mL Amp. Upon reaching an absorbance of *A*_600_ ~ 0.7, cells were harvested by fast filtration, flash-frozen in liquid nitrogen and lysed using Mixer Mill MM400 (Retsch) as described above for ribosome profiling. All following steps were carried out at 4 °C. The lysate was clarified by centrifugation at 20,000×*g* for 5 min and the supernatant (~1 mL) was desalted by gravity-flow gel-filtration through a 15 mL Sephadex G-25 column equilibrated in buffer containing 30 mM Hepes-KOH, pH 7.6, 100 mM KCl, 2 mM DTT, 0.5 mM PMSF, and 2 mM Mg(OAc)_2_. The flow-through fractions with *A*_260_ ≥ 10 were combined, flash-frozen in liquid nitrogen, and stored at −80 °C.

Prior to using the lysate for in vitro transcription–translation assays, the endogenous mRNAs were removed by nuclease treatment. For that, 50 µL of the thawed lysate were supplemented with 1 mM CaCl_2_ and 3.5 units of micrococcal nuclease (Thermo Fisher Scientific) and incubated for 10 min at room temperature. The reaction was quenched by addition of 2 mM EGTA and the lysate was immediately used for in vitro protein expression. Reactions were carried out in a total volume of 15 µL containing 7.5 µL of the nuclease-treated lysate supplemented with the following components (listed with their final concentrations): 22 mM Hepes-KOH, pH 7.6, 5.5 mM magnesium glutamate, 120 mM potassium glutamate, 1.7 mM DTT, 1.5 mM ATP, 1.5 mM GTP, 1.5 mM CTP, 1.5 mM UTP, 25 mM creatine phosphate, 0.26 mg/mL creatine kinase, 80 µM of each amino acid except methionine, 0.6 µCi/µL of [^35^S]-l-methionine (specific activity 1175 Ci/mmol) and 50 µg/mL of T7 RNA polymerase purified according to Durniak et al.^94^. The reactions were initiated by addition of 0.1 µg of the DNA templates prepared as described below. The reaction products were analyzed in 16 % SDS gels. Gels were fixed with 5% perchloric acid, stained with Coomassie blue, dried, and exposed overnight to a phosphorimager screen. Radioactivity was visualized in a Typhoon scanner (GE Healthcare). The intensity of the bands was quantified using ImageJ^95^.

### Preparation of DNA templates for in vitro transcription/translation

The DNA templates for the coupled transcription/translation reactions contained the 5′-untranslated region of tobacco mosaic virus RNA (also known as Ω leader 5′-GUAUUUUUACAACAAUUACCAACAACAACAAACAACAAACAACAUUACAAUUACUAUUUACAAUUACA-3′)^96^, followed by the protein coding sequence of the analyzed gene, and ending with a poly-A tail. All the primers used for generation of the templates are shown in Supplementary Table 3. To generate the *SLT2* and *ZEO1* templates, the gene sequences were PCR-amplified from genomic DNA of *S. cerevisiae* strain NOY891 using the primers Ω-SLT2/SLT2-A30 and Ω-ZEO1/ZEO1-A30, respectively. One microliter of the PCR product was diluted 1:100 to then re-amplify it using the T7 + T7-Ω primers combined with reverse primer SLT2-A30, for the *SLT2* template, or with ZEO1-A30 primer for the *ZEO1* template. The *GFP* gene was PCR-amplified from the pJL1-sfGFP plasmid^97^ using primers Ω-sfGFP and sfGFP-reverse. The resulting PCR product was re-amplified using primers T7, T7-Ω, and sfGFP-A37. The mutant *GFP* templates were generated by introducing the desired mutations via cross-over PCR^98^ using the respective mutagenizing primers listed in Supplementary Table 3 in combination with the T7 and sfGFP-A37 primers. The PCR products were cloned in pUC18 plasmid and the presence of the desired mutations was verified by sequencing. The templates for transcription-translation reactions were generated by PCR from the corresponding plasmids using the primers T7 and sfGFP-A37.

### Reporting summary

Further information on research design is available in the Nature Research Reporting Summary linked to this article.

## Supplementary information

Supplementary Information

Reporting Summary

## Data Availability

The data that support this study are available from the corresponding authors upon reasonable request. The cryo-EM and associated molecular model for the *S. cerevisiae* 80S ribosome complexed with telithromycin is available from the EMDB (EMD-11951[https://www.ebi.ac.uk/pdbe/entry/emdb/EMD-11951]) and PDB (7AZY [10.2210/pdb7AZY/pdb]), respectively. Ribo-seq data have been deposited in the NCBI Gene Expression Omnibus (GEO) database under accession code GSE164275. Source data is provided with this paper.

## Source data

Source Data

## Code availability

The software used for the analysis of Ribo-seq data is available from https://github.com/guydoshlab/Yeastcode1.
