# Supplementary material for: Context-specific action of macrolide antibiotics on the eukaryotic ribosome
Source: Nat Commun. 2021 May 14;12:2803. doi: 10.1038/s41467-021-23068-1 (PMC8121947; doi:10.1038/s41467-021-23068-1)
Supplement: Supplementary file 1 — Supplementary Information [file 41467_2021_23068_MOESM1_ESM.pdf]

Context-specific action of macrolide antibiotics on the eukaryotic ribosome

Maxim S. Svetlov, Timm O. Koller, Sezen Meydan, Vaishnavi Shankar, Dorota Klepacki, Norbert Polacek, Nicholas R. Guydosh, Nora Vázquez-Laslop, Daniel N. Wilson, Alexander S. Mankin

SUPPLEMENTARY INFORMATION

Supplementary Table 1. The minimal inhibitory concentrations (MICs) of different macrolide antibiotics against *S. cerevisiae* carrying wild-type or G2058A mutant 25S rRNA. The MIC values are indicated in µg/mL. TEL – telithromycin, SOL – solithromycin, CET – cethromycin, ERY – erythromycin, AZI – azithromycin, TYL – tylosin, SPI – spiramycin.

[a)] highest achievable concentration

	TEL	SOL	CET	RU_{69874}	CEM_{103}	ERY	AZI	TYL	SPI
WT	>3000[a)]	1500	1500	>375 [a)]	>375 [a)]	>3000 [a)]	>3000 [a)]	>2000 [a)]	>2000 [a)]
G2058A	187	47	750	>375 [a)]	>375 [a)]	>3000 [a)]	>3000 [a)]	>2000 [a)]	>2000 [a)]

Supplementary Table 2. Statistics for the final model of the 80S-TEL complex

	Yeast TEL-80S complex (PDB ID 7AZY) (EMD-11951)
**Data collection**	
Microscope	Titan Krios
Detector	K2 DDC
Pixel size (Å)	0.822
Defocus range (µm)	-1.0 to -3.0
Voltage (keV)	300
Electron dose (e^-/Å^2)	1.0
FSC threshold	0.143
Initial particle images (no.)	329,333
Final particle images (no.)	153,893
**Model composition**	
Initial model used (PDB code)	6Q8Y
Protein residues	6127
RNA nucleotides	3440
Waters	3
**Refinement**	
Resolution (Å)	2.877
Map sharpening B factor(Å^2)	-10
**Validation: proteins**	
Poor rotamers (%)	0.14
Ramachandran outliers (%)	0.43
Ramachandran allowed (%)	8.40
Ramachandran favored (%)	91.17
Bad backbone bonds (%)	0.01
Bad backbone angles (%)	0.02
**Validation: RNA**	
Correct sugar puckers (%)	97.99
Good backbone conformations (%)	79.71
Bad bonds (%)	0.00
Bad angles (%)	0.02
**Scores**	
MolProbity	1.58
Clash score, all atoms	2.76

Supplementary Table 3. DNA primers used in the study

Primer	Nucleotide sequence of the primer [a)]
25S-2430	CCCTCTATGTCTCTTCACAATGT
T7	TAATACGACTCACTATAGGG
T7-Ω	TAATACGACTCACTATAGGGAGTATTTTTACAACAATTACCAACAACAACAAACAA CAAACAACATTACAATTACTATTTACAATTACAT
Ω-SLT2	AACAAACAACATTACAATTACTATTTACAATTACATATGGCTGATAAGATAGAGAGGCAT
SLT2-A30	5’-TTTTTTTTTTTTTTTTTTTTTTTTTTTTTTCTAAAAATATTTTCTATCTAATCCAAACTC
Ω-ZEO1	CAAACAACATTACAATTACTATTTACAATTACATATGTCTGAAATTCAAAACAAAGCTGA
ZEO1-A30	TTTTTTTTTTTTTTTTTTTTTTTTTTTTTTTTAGTTGAAAATGGAAGCAATTTTACGGTT
Ω-sfGFP	AACAACAAACAACATTACAATTACTATTTACAATTACATATGAGCAAAGGTGAAGAACTG
sfGFP-reverse	5’-GCAAAAAACCCCTCAAGACCCGTTTA
sfGFP-A37	TTTTTTTTTTTTTTTTTTTTTTTTTTTTTTTTTTTTTTTATTTTTCGAACTGCGGATGGC
sfGFP K_{107}A fwd	GATGGCAAATATGCAACGCGCGCCGTT
sfGFP K_{107}A rev	AACGGCGCGCGTTGCATATTTGCCATC
sfGFP P_{196}A fwd	GTGATGGTCCGGTGCTGCTGGCGGATAATCATTATCTGAGCACGC
sfGFP P_{196}A rev	GCGTGCTCAGATAATGATTATCCGCCAGCAGCACCGGACCATCAC

[a)] Primer sequence corresponding to the protein coding sequence is underlined

a

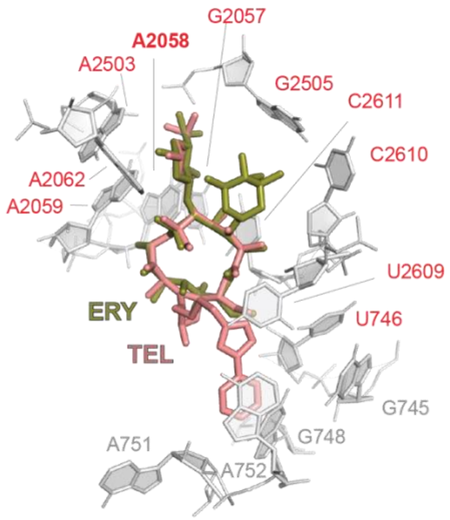

c

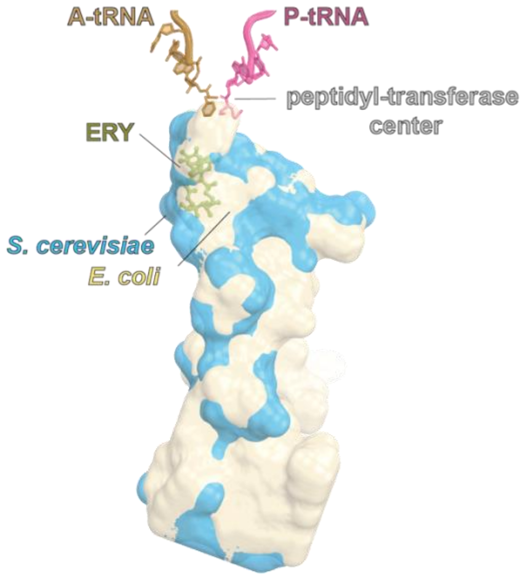

b

		745	746	748	751	752	2057	**2058**	2059	2062	2503	2505	2609	2610	2611
**Bacteria**	*Escherichia coli*	G	U	G	A	A	G	**A**	A	A	A	G	U	C	C
	Thermus thermophilus	G	A	G	A	A	A	**A**	A	A	A	G	U	C	U
	Staphylococcus aureus	G	U	G	A	A	G	**A**	A	A	A	G	U	C	C
	Streptococcus pneumoniae	G	U	G	A	A	G	**A**	A	A	A	G	U	C	C
	Bacillus subtilis	G	U	G	A	A	G	**A**	A	A	A	G	U	C	C
	Enterococcus faecalis	G	U	G	A	A	G	**A**	A	A	A	G	U	C	C
	Klebsiella pneumoniae	G	U	G	A	A	G	**A**	A	A	A	G	U	C	C
	Acinetobacter baumannii	G	U	G	A	A	G	**A**	A	A	A	G	U	C	C
	Mycobacterium tuberculosis	G	U	G	G	A	A	**A**	A	A	A	G	U	C	U
	Haemophilus influenzae	G	U	G	A	A	G	**A**	A	A	A	G	U	C	C
**Eukarya**	*Saccharomyces cerevisiae*	C	G	G	A	A	A	**G**	A	A	A	G	U	U	U
	Candida albicans	C	G	G	A	A	A	**G**	A	A	A	G	U	U	U
	Caenorhabditis elegans	C	G	G	A	A	A	**G**	A	A	A	G	U	U	U
	Mus musculus	G	G	U	A	U	A	**G**	A	A	A	G	U	U	U
	Bos taurus	A	G	G	A	A	A	**G**	A	A	A	G	A	U	A
	Canis lupus	G	G	U	A	C	A	**G**	A	A	A	G	U	U	U
	Felis catus	C	U	G	G	C	A	**G**	A	A	A	G	U	U	U
	Homo sapiens	G	U	G	G	A	A	**G**	A	A	A	G	U	U	U

Supplementary Figure 1. Variation of the rRNA nucleotide forming the macrolide-binding site. a. The 23S rRNA residues located within 5 Å of the bound erythromycin (ERY) or telithromycin (TEL) in the bacterial (*E. coli*) ribosome (PDB ID 4V7U and 4V7S [1], respectively). The nucleotides in contact with ERY and TEL are indicated in red and those in the vicinity of the TEL-specific arm are indicated in gray. **b.** The identity of 23S rRNA residues in the macrolide binding pocket in the bacterial and eukaryotic cytoplasmic ribosome. The nucleotide numbers and their coloring correspond to those in panel (**a**). **c.** The alignment of the NPET surfaces of the *E. coli* 70S (beige, PDB ID 4YBB [2]) and *S. cerevisiae* 80S (light blue, PDB ID 4V88 [3]) ribosomes. The molecule of a macrolide antibiotic (erythromycin) bound in the exit tunnel of the *E. coli* ribosome is colored salmon. The A- and P-site aminoacyl-tRNAs and their respective amino acids placed in the active site of the peptidyl transferase center are shown in brown and magenta, respectively.

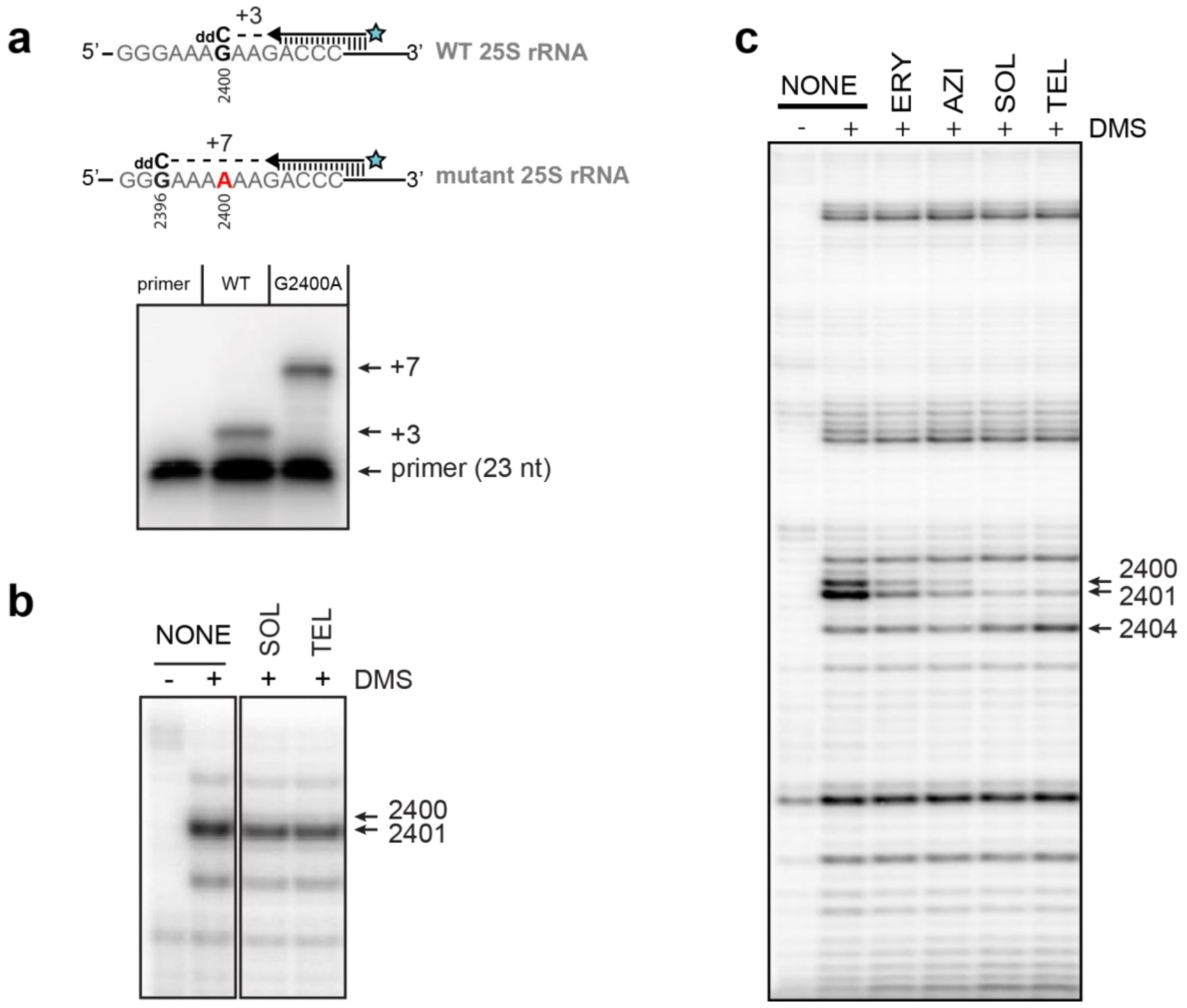

Supplementary Figure 2. Interaction of extended and non-extended macrolides with wt and mutant yeast ribosomes. a. Primer extension analysis demonstrating that the mutant yeast cells exclusively express the ribosomes with the G2400A mutation in the 25S rRNA. *Top*: the principle of primer extension analysis for assessing the ribosome population. In the presence of dATP, dGTP, dTTP, and ddCTP, the primer annealed to wild type (WT) 25S rRNA is extended by three nucleotides, whereas the primer annealed to the G2400A mutant 25S rRNA is extended by seven nucleotides. *Bottom*: separation of the primer extension cDNA products obtained using rRNA isolated from wild type or mutant G2400A cells in the 6% denaturing polyacrylamide gel. Shown is a representative gel of two independent experiments. **b.** DMS footprinting shows that in contrast to their binding to the G2400A mutant ribosome (**Fig. 1c**), SOL or TEL do not bind to the wt yeast ribosome. The primer extension bands corresponding to the 25S rRNA residues G2400 and A2401 are indicated by arrows. **c**. While the extended macrolides (TEL and SOL) efficiently protect A2400 and A2401 from DMS modification, ERY and AZI, that lack alkyl-aryl side chains found in TEL and SOL, afford only marginal protection indicating only weak binding. The uncropped gels and a gel with the reference sequence lanes can be found in the Source data file. Panels **a-c** show representative gels of two independent experiments.

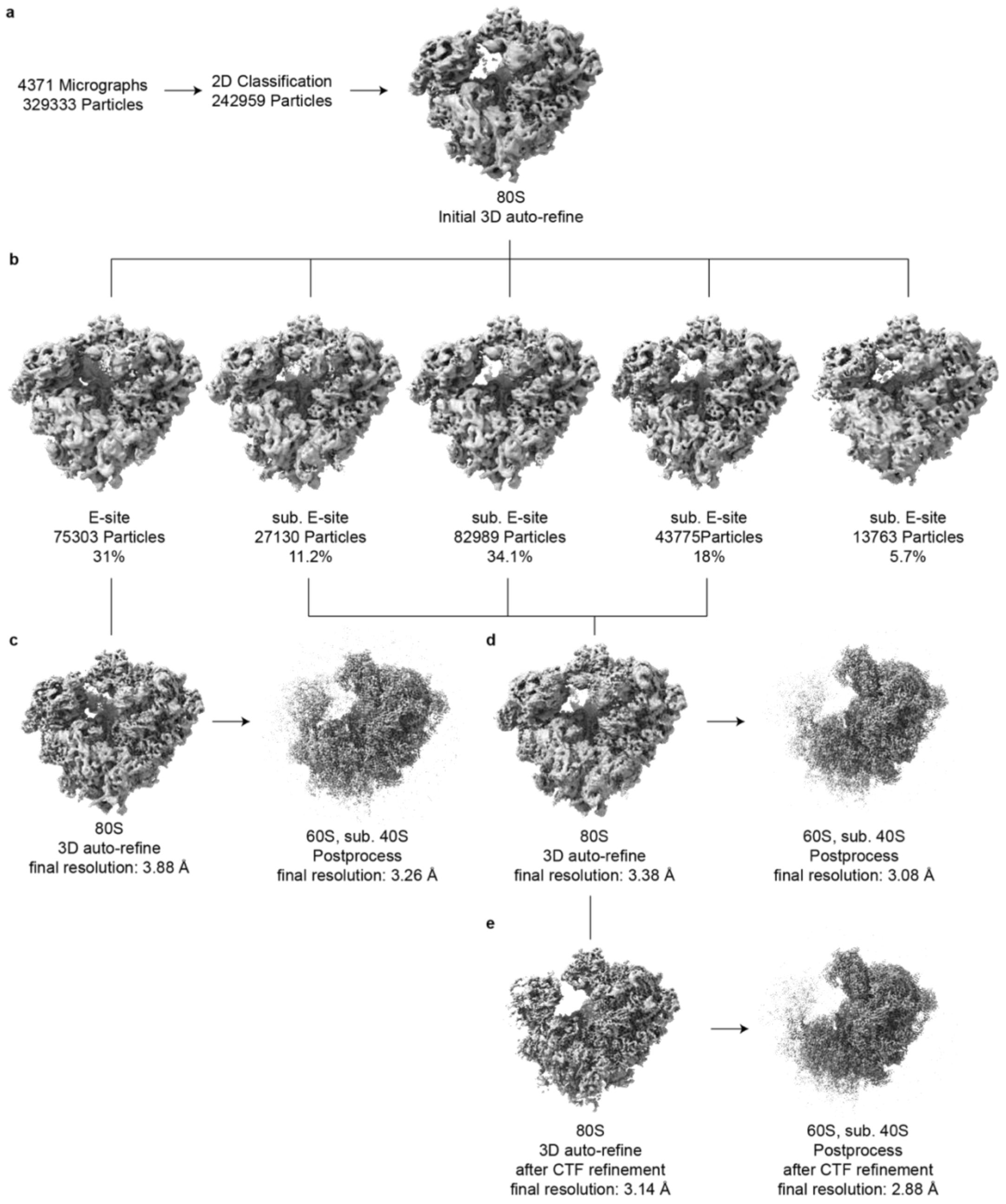

Supplementary Figure 3. *In silico* sorting of the cryo-EM data of 60S-TEL complex. a, A total of 329,333 particles were first subjected to 2D classification, resulting in 242,959 ribosome-like particles and an initial 3D reconstruction of an 80S ribosome. **b**, 3D classification (200 iterations) yielded five classes of structures. **c-d**, The particles containing either stoichiometric E-site tRNA (class 1, 75,303 particles) or sub-stoichiometric E-site tRNA (classes 2-4, 153,893 particles) were processed further independently. **e**, The highest resolution reconstruction of yeast mutant G2400A large 60S subunit with sub-stoichiometric small 40S subunit had an average resolution of 2.9 Å.

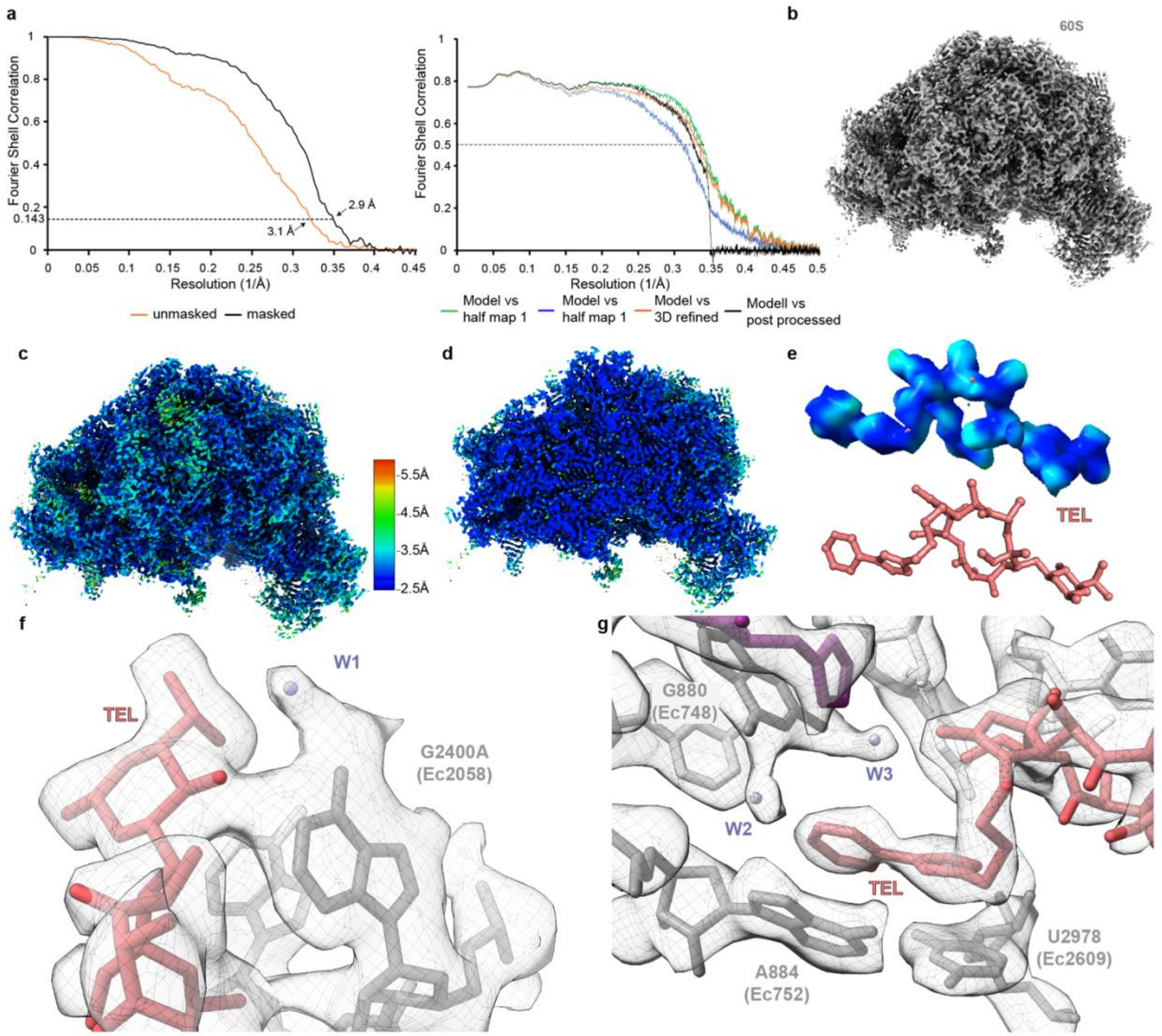

Supplementary Figure 4. Overview and resolution of the 60S-TEL-complex. **a**, Fourier Shell Correlation (FSC) curves indicating (left) that the final reconstruction of yeast mutant G2400A had an average resolution (at 0.143 FSC) of 3.1 Å (unmasked, orange) and 2.9 Å (masked, black) and (right) showing the map vs model cross correlation for half map 1 (green), half map 2 (blue), unmasked 3D refined (orange) and masked post processed volumes (black). **b**, Electron density of yeast mutant G2400A large 60S subunit. **c,d**, Cryo-EM map of 60S-TEL complex shown as (**c**) overview and (**d**) cut through, colored according to local resolution. **e**, (*top*) cryo-EM density for TEL in the 60S-TEL complex colored according to local resolution and (*bottom*) the fitted structure (salmon). **f**,**e**, Isolated electron densities (grey mesh) with fitted model for TEL (**f**) desosamine and (**g**) alkyl-aryl side chains (salmon), uL22 protein (purple), surrounding 25S rRNA nucleotides (grey) and water molecules W1, W2 and W3 (light blue).

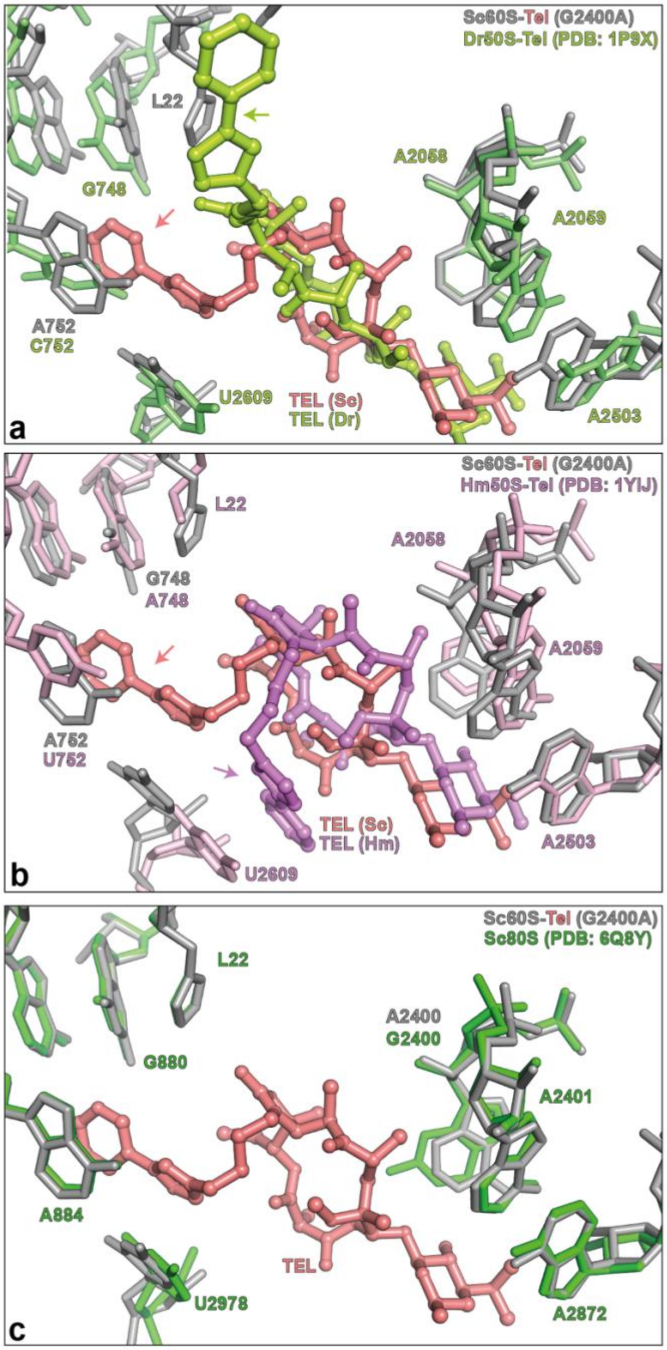

Supplementary Figure 5. Placement of the TEL alkyl-aryl side chain differs in the ribosomes of different species. a-c Superposition of the TEL-bound yeast mutant G2400A large 60S subunit with the structures of TEL-bound (**a,b**) or TEL-free (**c**) ribosomes of different species. In all the panels, the components of the mutant yeast ribosome are shown in grey and TEL is colored salmon. The 80S-TEL structure is aligned to (**a**) bacterial (*D. radiodurans*) 50S subunit complexed with TEL (green, PDB ID 1P9X [4]), (**b**) archaeal (*H. marismortui*) 50S subunit complexed with TEL (purple, PDB ID 1YIJ [5]), or (**c**) the vacant *S. cerevisiae* 60S subunit (green) (PDB ID 6Q8Y [6]).

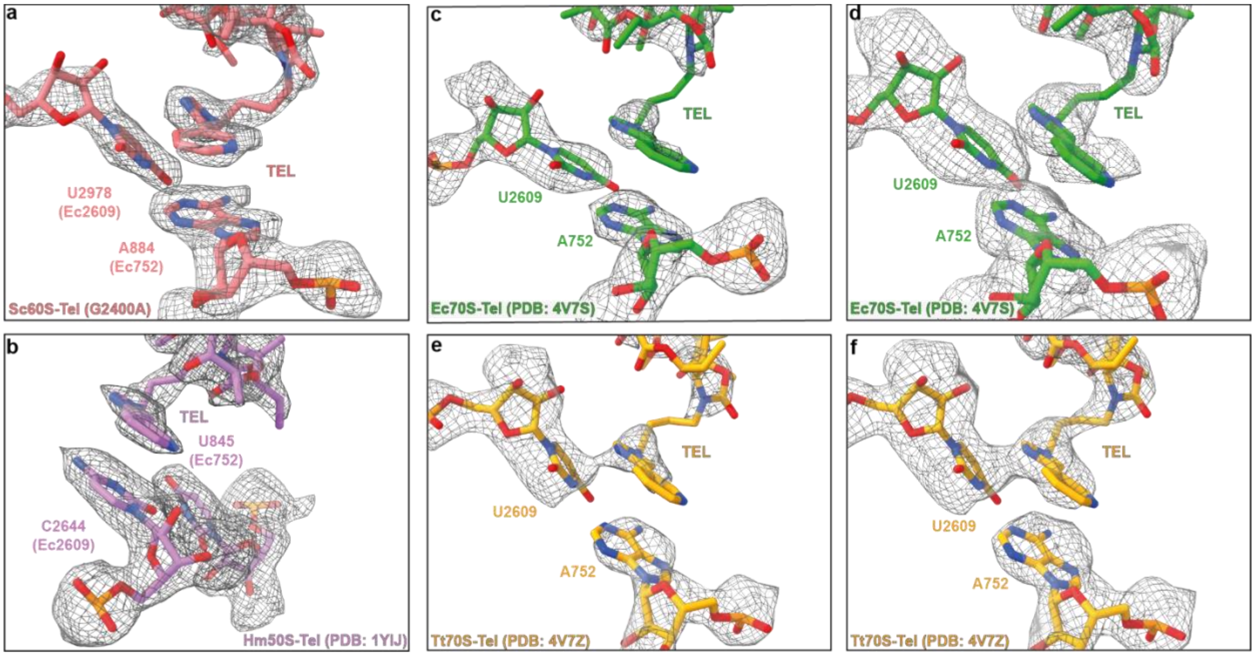

Supplementary Figure 6. Comparison of TEL alkyl-aryl ring molecular models from different species in their respective electron densities. a Molecular model of TEL in extracted electron density of yeast mutant G2400A with nucleotides A884 and U2978. **b** Molecular model of TEL in extracted electron density of H. marismortui with nucleotides U845 (Ec752) and C2644 (Ec2609) (PDBID: 1YIJ [5]). **c-d** Molecular model of TEL in extracted electron density of *E.coli* with nucleotides EcA752 and EcU2609 (PDBID: 4V7S [1]) at two different thresholds. **e-f** Molecular model of TEL in extracted electron density of *T.th* with nucleotides EcA752 and EcU2609 (PDBID: 4V7Z [7]) at two different thresholds.

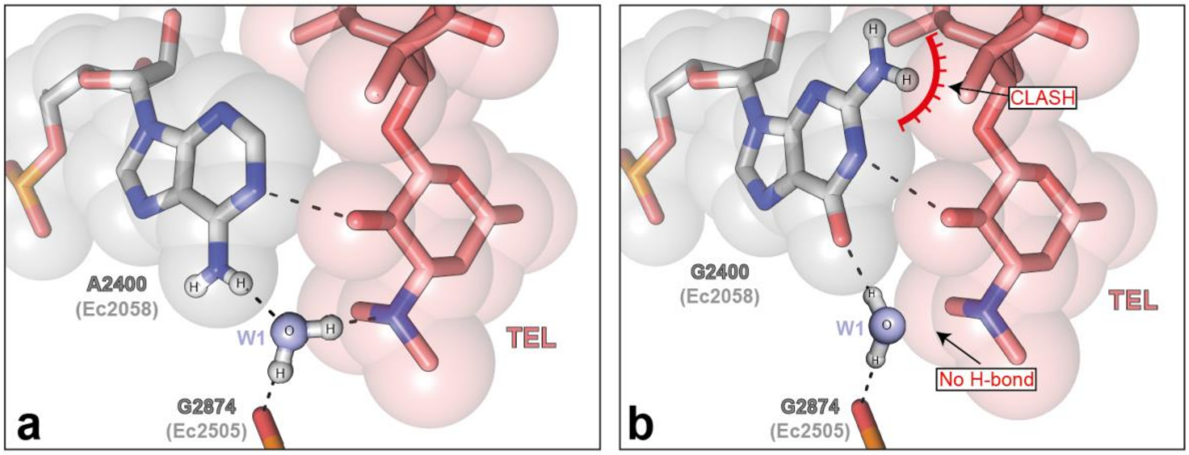

Supplementary Figure 7. The ability of TEL to act upon eukaryotic ribosome depends on the identity of the 25S rRNA residue 2400. C5 desosamine of TEL fits snuggly against A2400(A2058) in the mutant yeast ribosome (**a**) but would clash with G2400 in the wt yeast 60S subunit (**b**). In addition, the presence of guanine in this position would prevent water-bridged interactions of desosamine with rRNA observed in the G2400A mutant ribosome.

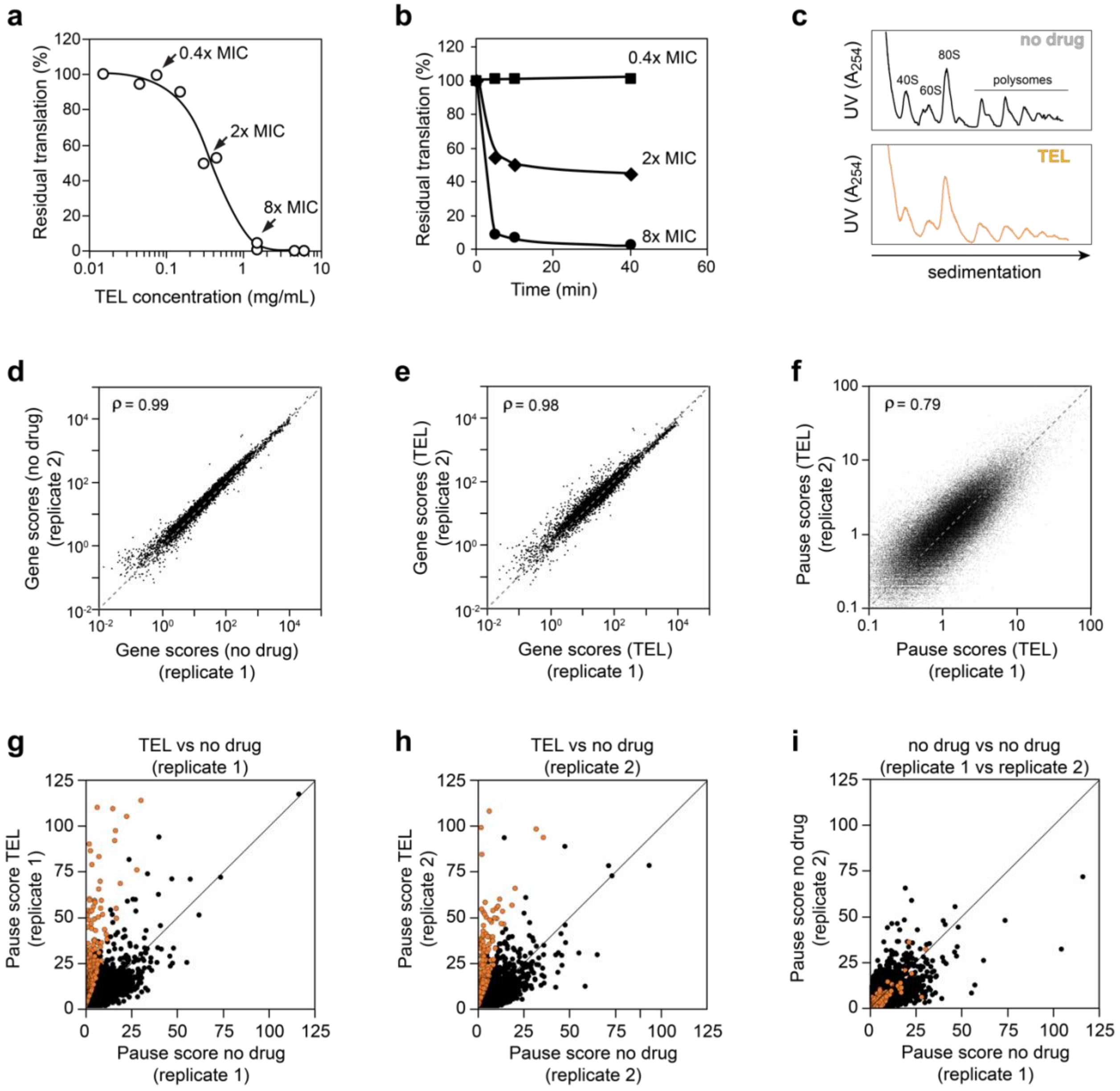

Supplementary Figure 8. Ribo-seq analysis of TEL-treated yeast cells. a, Residual protein synthesis in G2400A mutant yeast exposed for 10 min to varying concentrations of TEL. **b**, Time course of inhibition of translation in the G2400A mutant yeast cells exposed to 75 µg/mL (0.4X MIC), 375 µg/mL (2X MIC), or 1.5 mg/mL (8X MIC) of TEL. **c,** Polysome profile of untreated (‘no drug’) yeast cells and cells exposed for 10 min to 8x MIC of TEL. **(d-f),** Comparison of Ribo-seq gene scores in untreated cells (**d**) and TEL-treated cells (**e**) in two independent Ribo-seq experiments (5528 genes total). **f,** Comparison of pause scores at 46445 genomic sites in two independent Ribo-seq experiments in TEL-treated cells. ρ values represent Pearson correlation coefficient. **(g,h)**, Comparison of pause scores in TEL-treated vs untreated cells in two independent biological replicates (note that axes are not logarithmic as in (**d-f**), but linear: in order to facilitate comparison). 749 sites that show at least 2.5-fold increase in pause scores in TEL-treated cells in comparison with the untreated control in two independent experiments are highlighted in orange. **i**, same as **g** and **h**, but comparing pause scores in two untreated controls. Orange dots mark the same sites that are highlighted in **g** and **h**.

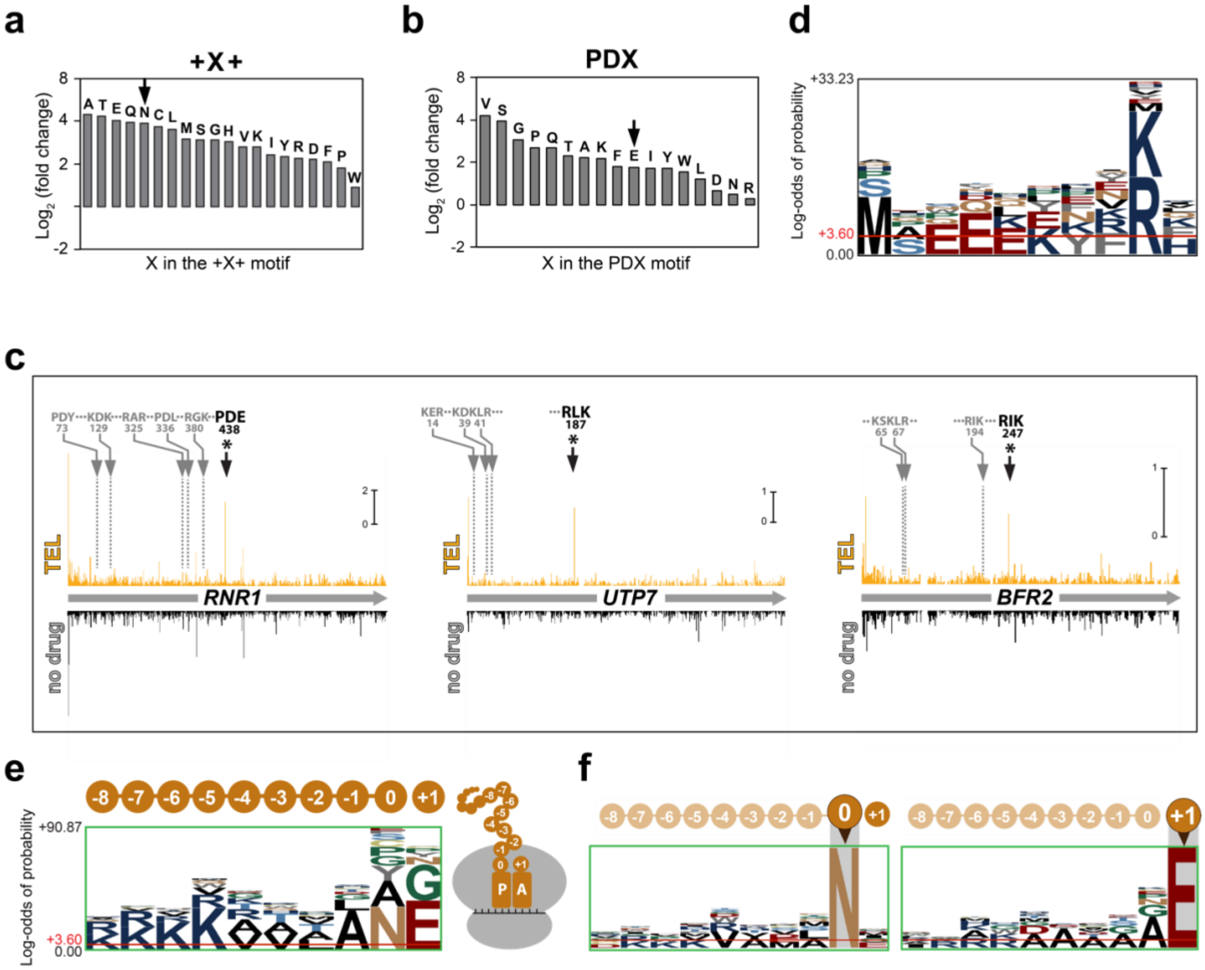

Supplementary Figure 9. Analysis of TEL translation arrest sites. a, The identity of the P-site amino acid influences the extent of TEL-mediated translation arrest at the +X+ motif. **b**, The influence of the A site amino acid on the efficiency of TEL-mediated translation arrest at the PDX motif. The y axes in panels (**a**) and (**b**) show $\log_2$ of median pause score difference in the TEL-treated vs. control samples. **c**, The +X+ or PDX motifs are required but not sufficient for TEL-mediated translation arrest in the yeast cell. Grey arrows indicate sites conforming to +X+ or PXX motifs where TEL does not induce ribosome stalling: compare density of ribosome footprints in the untreated control (grey) to that in the TEL-treated cells (orange). Note that TEL-mediated translation arrest occurs at specific +X+ or PDX motifs located farther downstream in the same genes, as evidenced by the associated density peaks (black arrows). **d,** pLogo analysis of 457 strong TEL translation arrest sites that do not conform to the +X+ or PDX motifs (related to panels (**b**) and (**h**) of main **Fig. 3**). **d**, pLogo analysis of amino acid sequences at 1809 genomic sites of the least efficient TEL action, where ribosome occupancy was reduced by more than 2-fold to the control in TEL-treated cells (the green box in the plot of main **Fig. 3b**). **f,** subset of the sites from (**e**), where (left) Asn (N) occupies the P site or (right) Glu (E) is placed in the A site. Noteworthy that neither a C-terminal Asn within the +X+ stalling motif nor a Glu residue in the PDX arrest sequence are able to counteract the TEL action (arrows on panels **a** and **b**).

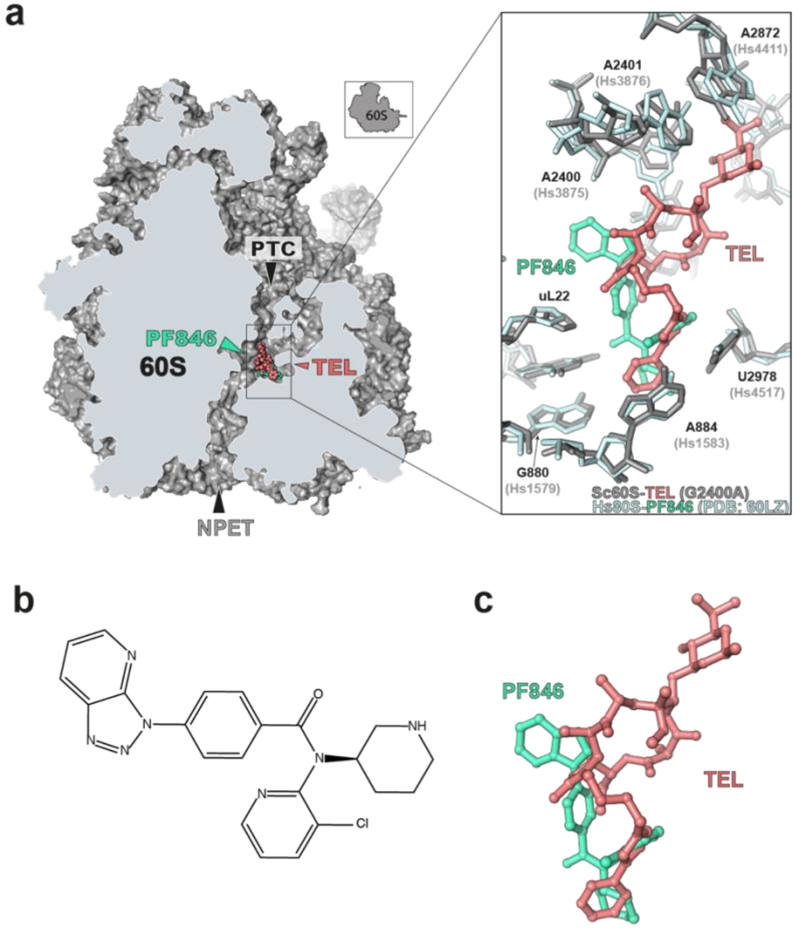

Supplementary Figure 10. Comparison of TEL binding within the yeast mutant G2400A large 60S subunit and PF846 binding to *H. sapiens* large 60S subunit. a, Transverse section of the large 60S subunit of the yeast mutant G2400A ribosome with molecular model of TEL (salmon) in the NPET and superimposed structure of PF846-80S structure (light green, PDB ID 6OLZ [8]). In the close-up view, rRNA and r-protein residues in proximity to TEL in the yeast ribosome TEL are shown in grey, and the residues approaching PF846 in the *H. sapiens* ribosome and shown in cyan. **b**, Chemical structure of PF846. **c**, Superposition of PF846 (light green,) and TEL (salmon) in their respective eukaryotic ribosome-bound states.

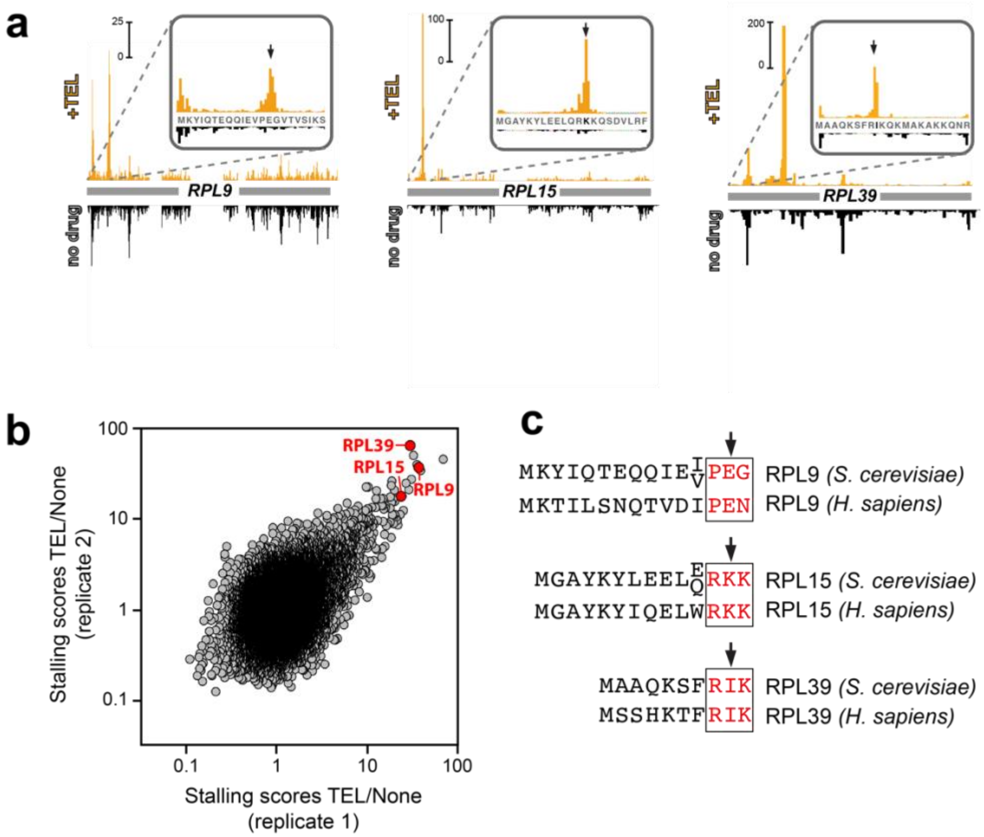

Supplementary Figure 11. Some of the strongest sites of TEL-induced translation arrest are in the genes of ribosomal proteins associated with cancer. a, Sites of TEL-induced ribosome stalling in the yeast ribosomal protein genes RPL9, RPL15 and RPL39. **b**, The stalling sites within the three ribosomal protein genes in panel (a) are among the sites of the most pronounced TEL-induced arrest genome-wide. The corresponding sites are shown in red on the general pause score graph taken from Fig. 4b. **c**, Conservation of the sites of TEL-induced arrest between ribosomal protein genes in yeast and human cells.

Supplementary References

1. Dunkle, J.A., Xiong, L., Mankin, A.S. & Cate, J.H. Structures of the *Escherichia coli* ribosome with antibiotics bound near the peptidyl transferase center explain spectra of drug action. *Proc Natl Acad Sci USA* **107**, 17152-17157 (2010).
2. Noeske, J. et al. High-resolution structure of the *Escherichia coli* ribosome. *Nat Struct Mol Biol* **22**, 336-341 (2015).
3. Ben-Shem, A. et al. The structure of the eukaryotic ribosome at 3.0 A resolution. *Science* **334**, 1524-1529 (2011).
4. Berisio, R. et al. Structural insight into the antibiotic action of telithromycin against resistant mutants. *J Bacteriol* **185**, 4276-4279 (2003).
5. Tu, D., Blaha, G., Moore, P.B. & Steitz, T.A. Structures of MLSBK antibiotics bound to mutated large ribosomal subunits provide a structural explanation for resistance. *Cell* **121**, 257-270 (2005).
6. Tesina, P. et al. Structure of the 80S ribosome-Xrn1 nuclease complex. *Nat Struct Mol Biol* **26**, 275-280 (2019).
7. Bulkley, D., Innis, C.A., Blaha, G. & Steitz, T.A. Revisiting the structures of several antibiotics bound to the bacterial ribosome. *Proc Natl Acad Sci USA* **107**, 17158-17163 (2010).
8. Li, W. et al. Structural basis for selective stalling of human ribosome nascent chain complexes by a drug-like molecule. *Nat Struct Mol Biol* **26**, 501-509 (2019).
